# Exploring the phenotypic diversity of *Eragrostis tef* for biomass and grain production under optimum growth conditions

**DOI:** 10.3389/fpls.2025.1538510

**Published:** 2025-03-17

**Authors:** Mitiku Asfaw Mengistu, Won Cheol Yim, Juan K. Q. Solomon, John C. Cushman

**Affiliations:** ^1^ MS330/Department of Biochemistry & Molecular Biology, University of Nevada, Reno, NV, United States; ^2^ MS202/Department of Agriculture, Veterinary & Rangeland Sciences, Reno, NV, United States

**Keywords:** *Eragrostis tef*, Tef, phenotyping, agronomic traits, biomass, grain, biofuel feedstock

## Abstract

**Introduction:**

Tef or Teff [*Eragrostis tef* (Zucc.) Trotter)] is a C_4_ photosynthesis tropical grass species within the Poaceae valued for its high-quality forage, fodder, and highly nutritious, gluten-free grain while showing great potential as a bioenergy crop due to its high biomass productivity and climate resilience. Here, we document the extensive phenotypic diversity of 368 *E. tef* accessions within the United States Department of Agriculture (USDA)-Agricultural Research Service (ARS) national germplasm collection.

**Methods:**

Two morphological (*e.g.*, panicle morphology and seed color) and 11 quantitative agronomic (*e.g.*, including fresh weight, dry weight, straw yield, seed yield, harvest index, plant height, panicle length, tiller count, floret count, hundred-seed weight, and seed area) traits were characterized under idealized growth conditions.

**Results:**

Five major panicle forms were identified including very loose, loose, semi-loose, compact, and, very compact, which were present in 24%, 25%, 25%, 18%, and 8% of accessions, respectively. Accessions with very compact panicles showed the highest biomass production and plant height, whereas accessions with loose and very loose panicle forms showed the highest tiller counts, seed yield, and harvest indices. White-seeded accessions were more numerous (55%) than brown-seeded accessions (45%) with white seeds being more common in very compact, compact, and semi-loose panicle forms and brown seeds being more common in loose and very loose panicle forms. Correlation analysis revealed positive associations among fresh weight, dry weight, straw yield, and plant height was well as seed yield and harvest index. Hundred-seed weight was positively correlated with seed area, plant height, and panicle length. Principal component analysis identified fresh weight, dry weight, and straw yield as major contributors (72.6%) to total trait variation. Hierarchical agglomerative clustering analysis revealed five distinct groups based upon the quantitative agronomic traits.

**Discussion:**

These data provide an invaluable resource for genome-wide association studies, stratified sampling, and parental line selection for ongoing *E. tef* breeding programs.

## 1 Introduction

Tef or Teff [*Eragrostis tef* (Zucc.) Trotter] is an allotetraploid, self-pollinated, annual warm season, C_4_-photosynthesis cereal within the Poaceae, sub-family Eragrostoidae (Assefa et al., 2011b, 2015). The *Eragrostis* genus contains about 350 species (Wartson and Dallwitz, 1992). However, *E. tef* is the only species within the tribe Eragrosteae and genus *Eragrostis* that is cultivated as a major cereal crop (Chanyalew et al., 2019). The species name for *E. tef* is derived from the Amharic word “t’efa’” meaning “lost” (Rouk and Hailu, 1963), an appropriate description of its tiny seeds, which are likely the smallest of the domesticated grain crops.

Ethiopia is the primary center of origin and diversity of *E. tef*, where the crop is believed to have been domesticated as early as 5,000 B.C (Vavilov, 1951; Mengesha, 1966; Belete, 2020). *E. tef* is an ancient grain cultivated extensively in Ethiopia and Eritrea where it serves as a major staple food used as flour for the production of *injera* for more than two-thirds of Ethiopia’s population (Assefa et al., 2011b, 2015; Dessie, 2018). *E. tef* is the most important Ethiopian cereal crop where it accounts for about 28% of the total acreage and 21% of the gross annual grain production (Central Statistical Authority, 2017). However, *E. tef* is also gaining global popularity as a high-quality forage, fodder, and grain crop (Barretto et al., 2021), in brewing and malting (Gebremariam et al., 2014), and as an emerging bioenergy feedstock (Nigatu et al., 2012; Chufo et al., 2015). The highly nutritious and gluten-free grain of *E. tef* with its balanced protein profile of essential amino acids, abundant dietary fiber, and dense mineral composition, particularly calcium, magnesium, and iron, has made it popular in many parts of the world including the USA, Israel, the Netherlands, and Australia (Bultosa, 2007; Zewdu and Solomon, 2007; Hager et al., 2012; Zhu, 2018). The grain is now in high demand for its highly nutritious, gluten (α-gliadin)-free flour suitable for consumption by gluten-intolerant individuals (Spaenij-Dekking et al., 2005).

In addition to grain production, *E. tef* is highly prized as a forage and fodder in South Africa, India, Pakistan, Uganda, Kenya, and Mozambique (Girma et al., 2014). Importantly*, E. tef* straw provides a highly palatable livestock fodder, fetches high market prices, and shows minimal loss due to pests upon storage (Ketema, 1993; Belete, 2020). *E. tef* is a versatile and resilient crop that grows well under extreme environmental conditions, such as drought and water-logging (Zhu, 2018; Chanyalew et al., 2019), and shows suitability in various cropping systems like crop rotation, intercropping, and double cropping (Ketema, 1997; Belete, 2020). Despite its resilience and adaptability to environmental factors, *E. tef* grain production remains relatively low in Ethiopia ranging from ~700 kg ha^-1^ to ~1700 kg ha^-1^ (Central Statistical Authority, 2005-2013; Chanyalew et al., 2019; Belete et al., 2020). However, more recent studies have identified seeding rates, seedling transplanting, and row planting and spacing strategies to improve grain yields to >2,300 kg ha^-1^ (Arefaine et al., 2020; Mihretie et al., 2021). Optimization of K fertilization rates (in the range of 60–90 kg ha^-1^) has also been shown to increase grain yield to >3,400 kg ha^-1^ depending upon field sites (Demiss et al., 2020). Optimal N and K fertilization rates of 92 kg ha^-1^ and 46 kg ha^-1^, respectively, were shown to increase grain yields to >2,000 kg ha^-1^ depending upon the landscape topology (Gedamu et al., 2023). The susceptibility of *E. tef* to lodging and extreme drought accounts for most of its production losses. Lodging accounts for 17-22% of yield losses annually and results in reduced grain and straw quality depending upon the variety and local conditions (Ketema, 1993; Assefa et al., 2011b; Paff and Asseng, 2019). Drought stress is estimated to reduce *E. tef* yield by an average of 40% and up to 77% if the stress occurs during anthesis (Abraha et al., 2015).

*E. tef* grain yield has improved by over 50% in the last two decades through conventional breeding and greater adoption of improved varieties and production practices (Chanyalew et al., 2019). An important part of these advances has been the ability to assess the available diversity of *E. tef* through carefully controlled varietal trials. For example, the phenotypic diversity across quantitative agronomic traits has been evaluated by several studies performed at various field locations within the major growing regions of Ethiopia (Adnew et al., 2005; Teklu and Tefera, 2005; Chanyalew et al., 2009; Tsige et al., 2018; Abewa et al., 2019; Girma et al., 2019; Jifar et al., 2020). An *E. tef* diversity panel of 273 accessions has also been evaluated for phenotypic traits in central and southern Israel (Ben-Zeev et al., 2018). Another study in Israel evaluated 13 *E. tef* accessions based upon a set of agronomic traits, including lodging, seed color, grain yield, average grain weight, and plant height, as well as sensory evaluation of *injera* produced from flour from these accessions grown under both greenhouse and field conditions (Merchuk-Ovnat et al., 2020). More recently, a collection of 317 accessions and three improved cultivars revealed a wide range of grain yields and harvest indices suggesting the existence of genetic potential to further improve *E. tef* grain yields (Bayable et al., 2021).

Broad genetic diversity is known to improve crop productivity and resilience in modern breeding programs (Swarup et al., 2021). Over 5,000 *E. tef* accessions are known to exist from diverse geographical regions of Ethiopia from different elevations and climatic conditions (Assefa et al., 2015). While yields of many cereal grains (*e.g.*, barley, maize, rice, wheat) will be negatively impacted by the effects of climate change (Challinor et al., 2014; Zhao et al., 2017; Leng and Hall, 2019), others such as *E. tef*, are less likely to be affected negatively (Gebresamuel et al., 2022). A huge genetic diversity exists for *E. tef* germplasm resources with over 3,850 accessions available within the Ethiopian Biodiversity Institute (EBI (https://www.ebi.gov.et/) collection representing a wide range of geographical and bioclimatic zones within Ethiopia (Woldeyohannes et al., 2020). However, modeling of *E. tef* performance using current climate projections predicts that suitable growing areas within Ethiopia will diminish by 2070 (Woldeyohannes et al., 2020). The genetic diversity of *E. tef* cultivars might suffer from narrow genetic diversity due to the repeated use of relatively few parental lines (*e.g.*, ‘*Dukem’*, ‘*Magna’*, ‘*Tseday’*, ‘*Quncho’, etc.*) in modern breeding programs, especially in Ethiopia (Assefa et al., 2015). Greater investments in breeding efforts are needed to improve tef cultivars (Tadele et al., 2024). The overall goal of this study was to quantify the extent and pattern of existing phenotypic variability among 368 USDA-ARS *E. tef* accessions, mostly from Ethiopia, and to identify accessions useful for such improvement efforts.

## 2 Materials and methods

### 2.1 Seedling establishment, trial management, and greenhouse conditions

A total of 368 *Eragrostis tef* (Zucc.) Trotter (Poaceae) accessions were obtained from the United States Department of Agriculture (USDA)-Agricultural Research Service (ARS), Western Region Plant Introduction Station, Washington State University, Pullman, WA. Of these, one accession was determined to be *Eragrostis curvula*. An additional named cultivar called ‘*Dessie*’ was used as a control. Therefore, a total of 368 *E. tef* accessions were evaluated. Two to three seeds of each accession were planted in 20.3 cm pots with a volume of 3.8 L filled with a standard soil mix (Metromix^®^ 200 soil mix, Sun Gro Horticulture, Bellevue, WA). After two weeks, plants were thinned to maintain only one seedling per pot. Thus, a single seed was used to maximize uniformity when performing each replicate study. Plants were supplied with slow-release fertilizer following the recommended application rates (Osmocote^®^ Smart-Release^®^ Plant Food Plus, The Scotts Company, Marysville, OH). Powdery mildew was controlled by daily vaporization of elemental sulfur (Duda Energy, LLC, Decatur, AL) using a commercial sulfur vaporizer (Grower’s Edge, Inc., Johnston, IA). Aphid infestation was observed on occasion but was controlled with the application of Acephate 97UP (Orthene^®^ in granular and aerosol forms, United Phosphorous, Inc., King of Prussia, PA) according to manufacturer’s instructions (2 g per pot every six months or 2 g per 4 L water for aerosol application repeated every two weeks as needed). The greenhouse conditions were maintained throughout the growing season with natural lighting limited to 2,000 µmole m^-2^s^-1^ light by shade cloth, mean daily temperature of 26°C and 18°C during the day and night, respectively, and irrigation occurring daily for 15 minutes from an overhead mist system.

### 2.2 Experimental design, data collection, and analysis

The 368 *E. tef* accessions, including ‘*Dessie*’ as a control, were laid out in a randomized complete block design (RCBD) with each of the three replications conducted separately over three years during the same summer growing season (June-November). The layout consisted of three blocks or replications. Each accession was assigned randomly to a different position within each block for each replicate to minimize possible variations in conditions within the greenhouse. However, the conditions within the greenhouse were very uniform as the light, temperature, and watering conditions were tightly controlled for each replication. While standard evaluation systems are in place for major crops like rice (Biodiversity International, 2007), barley (Cross et al., 1992), and wheat (IBPGR, 1985), such standardized systems of descriptors have not yet been established for *E. tef*. However, the National *E. tef* Improvement Program in Ethiopia uses a set of agronomic parameters as morphological markers to evaluate *E. tef* genotypes (Assefa et al., 2015). Thus, the following two morphological parameters and 11 quantitative agronomic traits were used to evaluate the accessions.

#### 2.2.1 Panicle morphology

Panicle morphology was based upon panicle features including pattern and mode of branching, degree of openness and angle of primary branches, and spikelet density, and organized into very compact, compact, semi-loose, loose and very loose groups.

*Seed color:* Seed color was determined through visual observation and images were captured using a Nikon SMZ800 microscope (Nikon Imaging Products, Melville, NY, U.S.) and Nikon Coolpix 4300 camera (Nikon Imaging Products). ImageJ software was used to analyze the images and determined the color of the seed.

#### 2.2.2 Fresh weight

Fresh weight (FRW) of the total fresh above-ground biomass per plant in grams (g). Weight of total fresh biomass was measured immediately after harvesting using an Ohaus Explorer^®^ Precision Balance (Ohaus Corporation, Inc., Parsippany, NJ, USA). The above-ground biomass was comprised of the stem, leaf sheaths, leaf blades, and the seed-bearing inflorescences.

#### 2.2.3 Dry weight

Dry weight (DRW) was measured after drying the fresh biomass at 70°C for 72 h using a drying oven (DKN812C, Yamato Scientific America, Inc., Santa Clara, CA) and weighing the material using an Ohaus Explorer^®^ Precision Balance.

#### 2.2.4 Straw yield

Total dry weight of straw yield (STY) representing the above-ground biomass excluding seed yield per plant measured in grams (g) after oven drying of samples. Straw yield measurements were collected using an Ohaus Explorer^®^ Precision Balance.

#### 2.2.5 Seed yield

The seed yield (SDY) per plant was obtained from each accession in grams (g) using an Ohaus Adventurer Pro^®^ Precision Balance.

#### 2.2.6 Harvest index

Harvest index (HI) was calculated as a ratio of seed yield to total dry biomass.

#### 2.2.7 Plant height

The length of the main tiller or stem (PLH) was measured from the crown, where the plant stem meets the roots, to the tip of the panicle at maturity in centimeters (cm) and represents the sum of the measures of culm height and panicle length.

#### 2.2.8 Tiller count

The total number of tillers per plant (TLC) was counted manually when the plants had reached at least 75% maturity.

#### 2.2.9 Panicle length

The length of the main axis of the panicle (PNL) was measured from panicle base to the tip of the panicle in centimeters (cm) using a Stanley^®^ Fatmax^®^ Classic Tape Measure (Stanley Tools, Inc., New Britain, CT). For each replication, the average of five representative panicles per plant was recorded. Panicle length measurements were collected at full plant maturity.

#### 2.2.10 Floret count per spikelet

The number of florets per spikelet (FTC) present on fully mature plants was hand-counted during harvesting. Each replication was the average number of florets per spikelet counted from five selected panicles of a given accession. In *E. tef*, the number of florets per spikelet generally decrease as one moves from the tip to the bottom of the panicle. To avoid sampling bias, each selected panicle was uniformly sampled at the top, middle, and bottom sections of the panicle. Data were collected at full plant maturity.

#### 2.2.11 Seed area

ImageJ software (Schneider et al., 2012) was used to measure seed area (SDA) and other dimensions including the length, width, and circularity of each seed. Images were captured using a Nikon SMZ800 microscope (Nikon Imaging Products, Melville, NY, U.S.) and Nikon Coolpix 4300 camera (Nikon Imaging Products). Each replication was represented by 20 fully developed seeds.

#### 2.2.12 Hundred-seed weight

The weight of 100 well-developed randomly selected seeds (HSW) was collected for each accession. The weight of 100 seeds was measured using an analytical mass balance (Model 100L 2524T, precision = 0.001 g, Adam Equipment, Inc., Oxford, CT).

### 2.3 Data analysis

Accessions were evaluated under greenhouse conditions for two qualitative morphological parameters and 11 quantitative agronomic traits with three replications. Data were subjected to various statistical analyses including analysis of variance (ANOVA), principal component analysis, cluster analysis, correlation and regression analysis using R and Prism (GraphPad Software, Inc.) statistical software. Means of the three measurements of each accession were used in principal component, correlation, and cluster analyses, whereas individual replication measurements were used for ANOVA. R packages including ‘FactoMineR’ (Lê et al., 2008), ‘cluster’ (Maechler et al., 2013), and ‘factoextra’ (Kassambara and Mundt, 2017) were used in the multivariate analyses and for the visualization of results. Blocks were the random effect, while accessions (or genotypes) were fixed effects. Range, coefficient of genotypic and phenotypic variances, and broad-sense heritability were computed following standard procedures using the R package TraitStat (Nitesh et al., 2020).

In the RCBD, blocking and replications were the main sources of variability accounted for by the design. Range refers to the difference between the highest and lowest values of a quantitative trait. R = V_h_ – V_l_; where R is range, V_h_ and V_l_ are the highest and lowest values, respectively. Genotypic, environmental, and phenotypic variances and genotypic/phenotypic coefficients of variation were estimated according to (Zaki and Radwan, 2022) as:


Genotopic variance (σg2)=(MSg− MSe)/r Environmental variance (σe2)=MSe/rPhenotypic variance (σp2)=σg2+ σe2Genotypic coefficient of variation =Genotypic VarianceMean×100Phenotypic coefficient of variation =Phenotypic VarianceMean×100

Heritability in the broad sense was computed as the ratio of the percentage of genotypic variance to phenotypic variance according to (Zaki and Radwan, 2022) as:


$$Heritability\;\left(broad\;sense\right)\;=\frac{Genotypic\;Variance}{Phenotypic\;Variance}\times 100$$

Genetic advance as a percent of mean (GAM) was estimated as


$$GAM\%\;=\frac{K*H*p}{Mean}\times 100$$

Where:

K = 2.06 at 5% selection intensity.

H = Heritability.

P = Phenotypic standard deviation.

Genotypic and phenotypic coefficients of variance and heritability were computed using TraitStat, an open source R package (Nitesh et al., 2020). Genotypic and phenotypic coefficients of variance (GCV, PCV) and genetic advance (GA) and genetic advance as a percent of mean (GAM) were classified into three classes as follows: less than 10% (Low), 10–20% (Moderate), and more than 20% (High) (Zaki and Radwan, 2022). Heritability (h2) were classified into three classes as follows: 0.0-30% low (L), 31-60% medium (M) and > 60% high (H).

ANOVA, *t*-test, and subsequent mean separation procedures were computed using GraphPad Prism (Version 10.4.0) software. Pearson correlation coefficient analyses were computed to examine the degree and directions of associations between quantitative traits. The R packages mentioned above were used for analyses and visualization of results from correlation analyses, principal component, and hierarchical agglomerative clustering. Data were also visualized using GraphPad Prism (Version 10.4.0) software.

## 3 Results

### 3.1 Overview of morphological and agronomic trait diversity

**Figure 1:**
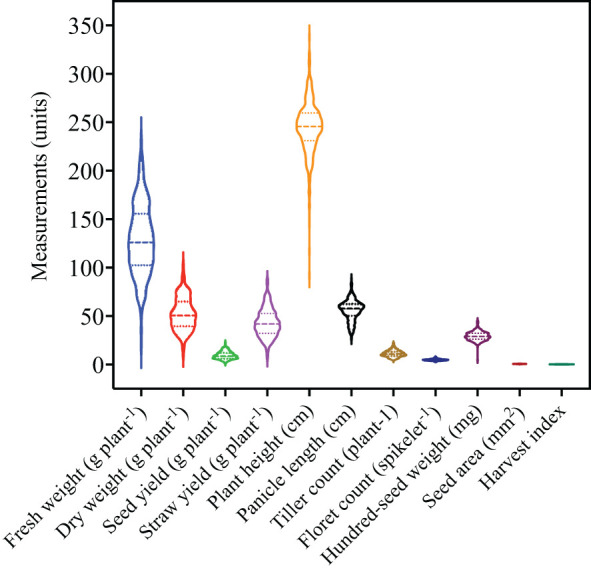
Overview of quantitative trait variability among 368 *E. tef* accessions. The accessions were evaluated for 11 quantitative traits including fresh weight, dry weight, seed yield, straw yield, plant height, panicle length, tiller count, floret count, hundred-seed weight, seed area, and harvest index. Violin plots show the distribution and density of the traits within each data set. Wider sections of the violin plots represent a greater frequency of accession with a particular value. The center dashed line represents median of the distribution whereas the fine dotted lines represent the lower (first) and upper (third) quartile of each distribution, respectively.

Violin plots were used to visualize the distribution and density of each trait given the large number of accessions evaluated in the current study (**Figure 1**). Traits with the highest degree of variation included fresh weight, dry weight, straw yield, plant height, and panicle length. In contrast, less variation was observed for seed yield, tiller count, floret count, hundred-seed weight, seed area, and harvest index. ANOVA reveals significant differences among the accessions for the 11 agronomic traits (**Table 1**). The genetic parameters including phenotypic coefficient of variance, genotypic coefficient of variance, heritability, genetic advance, and genetic advance percent of mean were calculated for each trait (**Table 2**).

**Table 1:** Analysis of variance (ANOVA) revealed significant differences among accessions for 11 quantitative agronomic traits.

	FRW (g)	DRW (g)	SDY (g)	STY (g)	HI (ratio)	PLH (cm)	PNL (cm)	TLC (#)	FTC (#)	HSW (#)	SDA (mm^2^)
Replication	3	3	3	3	3	3	3	3	3	3	3
Treatment	368	368	368	368	368	368	368	368	368	368	368
TMSS^1^	4189.2 ****	897.0 ****	47.2 ****	632.8 ****	0.01 ****	2401.4 ****	338.3 ****	33.5 ****	2.4 ****	64.5 ****	0.02 ****
EMSS^2^	1801.0	356.0	20.2	245.5	0.003	519.2	86.0	12.3	1.2	9.2	0.01
Mean	129.1	52.6	9.6	42.9	0.18	244.4	56.0	11.28	5.0	29.3	0.62
Minimum	6.3	3.6	0.5	1.7	0.02	78.7	10.2	1.0	2.0	2.9	0.13
Maximum	393.3	162.9	43.9	138.6	0.7	365.8	104.1	32.0	11.0	47.4	1.36
CV	32.9	35.9	46.8	36.5	28.8	9.3	16.6	31.05	22.3	10.3	15.1
StdError (m)	24.5	10.9	2.6	9.1	0.03	13.2	5.4	2.02	0.6	1.8	0.05
StdError (d)	34.6	15.4	3.7	12.8	0.04	18.6	7.6	2.86	0.9	2.5	0.08

^1^Treatment Mean Sum of Squares (TMSS). ^2^Error Mean Sum of Squares (EMSS).

ANOVA was used to test the statistical significance of the effect of accessions (*n* = 368) on the 11 quantitative agronomic traits. The traits were fresh weight (FRW), dry weight (DRW), seed yield (SDY), straw yield (STY), harvest index (HI), plant height (PLH), panicle length (PNL), tiller count (TLC), floret count (FTC), hundred-seed weight (HSW), and seed area (SDA). All the measurements were taken on single-plant basis. Significance was declared at *P*<0.0001 (****).

**Table 2:** Phenotypic and genotypic coefficient of variance, heritability, and genetic advance estimates.

	GCV	PCV	h2	GA	GAM
Fresh weight (g)	21.86 H	39.4832 H	30.653 M	32.1798 H	24.9318 H
Dry weight (g)	25.5549 H	44.0736 H	33.6196 M	16.0389 M	30.5238 H
Seed yield (g)	31.1806 H	56.2413 H	30.7368 M	3.4222 L	35.6107 H
Straw yield (g)	26.478 H	45.1027 H	34.4641 M	13.7413 M	32.0211 H
Harvest index(ratio)	20.0099 H	35.0882 H	32.5215 M	0.0429 L	23.5071 H
Plant height (cm)	10.2499 M	13.8566 M	54.7173 M	38.1681 H	15.6188 M
Panicle length (cm)	16.3699 M	23.2767 H	49.4597 M	13.2879 M	23.7159 H
Tiller count (#)	23.6213 H	39.0103 H	36.6648 M	3.3219 L	29.4643 H
Floret count (#)	13.1481 M	25.8806 H	25.8092 L	0.6724 L	13.7599 M
HSW (#)	14.6593 M	17.9338 M	66.8159 H	7.2327 L	24.6842 H
Seed area (mm^2^)	8.1249 L	17.106 M	22.5598 L	0.0495 L	7.9497 L

Genotypic and phenotypic coefficient of variances, broad sense heritability, genetic advance, and genetic advance percent of mean were computed for the 11 quantitative agronomic traits of *E. tef* accessions (*n* = 368). Genotypic coefficient of variance (GCV) phenotypic coefficient of variance (PCV), heritability (h2), genetic advance (GA), and genetic advance percent of mean (GAM). The categories used for GCV, PCV, GA, and GAM were the same as those reported by Zaki and Radwan (2022) for three classes:<10% low (L), 10–20% medium (M), and >20% high (H). The categories used for h2 were the same as those reported by Zaki and Radwan (2022) for three classes: 0.0-30% low (L), 31-60% medium (M) and > 60% high (H).

### 3.2 Panicle morphology

**Figure 2:**
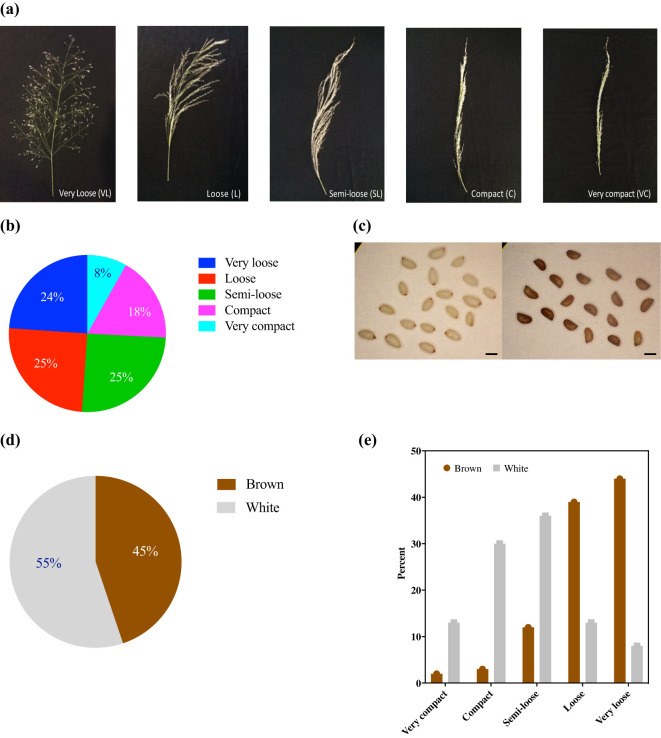
Five major panicle forms and two major seed colors identified among 368 *E. tef* accessions. **(a)** Representative images of each panicle form identified as very loose, loose, semi-loose, compact, and very compact. **(b)** Distribution of the panicle forms by percentage. **(c)** Representative images of white (ivory) (PI-196851) (left panel) and brown (PI-358594) seeds (right panel). Size bar = 1 mm. **(d)** Distribution of seed color (brown or white) by percentage. **(e)** Seed color (brown or white) and panicle form.

**Figure 3:**
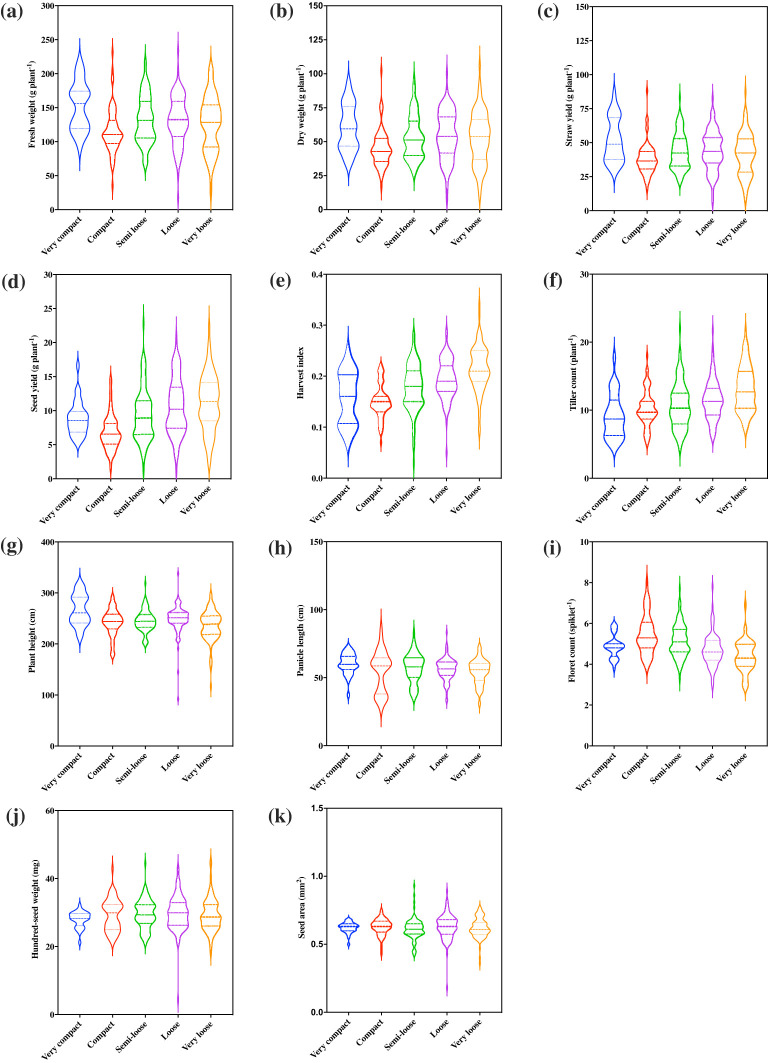
Association of panicle morphology with 11 quantitative agronomic traits among 368 *E. tef* accessions. **(a)** fresh weight of biomass, **(b)** dry weight of biomass, **(c)** straw yield, **(d)** seed yield, **(e)**, harvest index, **(f)** tiller count, **(g)** plant height, **(h)** panicle length, **(i)** floret count, **(j)** hundred-seed weight, and **(k)** seed area. Violin plots show the distribution and density of the traits within each data set. Wider sections of the violin plots represent a greater frequency of accession with a particular value. The center dashed line represents median of the distribution whereas the fine dotted lines represent the lower (first) and upper (third) quartile of each distribution, respectively.

Of the five representative panicle forms identified, very loose, loose, and semi-loose panicles were most prevalent and constituted 24%, 25%, and 25%, respectively, whereas the compact and very compact panicles constituted only 18% and 8%, respectively (**Figure 2**). Panicle morphology was associated with differences among the 11 quantitative agronomic traits. Violin plots revealed that panicle morphology was associated with key performance parameters and the relative distribution of accessions for each trait (**Figure 3**). Notably, accessions with loose and very loose panicle morphology showed the highest seed yield, whereas compact and very compact panicles showed the lowest seed yields (**Figure 3d**). In contrast, accessions with semi-loose, loose, and very loose panicles showed the highest harvest indices (**Figure 3e**) and the highest tiller counts (**Figure 3f**). Very compact panicle morphologies were also associated with higher above ground fresh and dry weight biomass, straw yield, and plant height (**Figure 3a, b, c, g**). Density plot graphs of the distributions of the 11 quantitative agronomic traits also revealed in greater detail how these traits were associated with panicle morphology (**Supplementary Figure S1**). Notably, accessions with very loose panicles showed higher but broad seed yield distributions, harvest indices, and tiller counts. In contrast, accessions with compact panicles displayed narrow distribution ranges for biomass traits, harvest index, seed yield, and tiller and floret counts. Furthermore, accessions with very compact panicles displayed narrow distribution ranges for seed yield, hundred-seed weight, seed area and tiller counts. Accessions with very compact panicles showed a bimodal distribution for above-ground fresh weight and straw yield with a clear peak associated with highest biomass and straw production (**Supplementary Figure S1**). Statistical analysis confirmed the effect of panicle morphology on quantitative agronomic traits including fresh weight, dry weight, seed yield productivity, straw yield productivity, harvest index, plant height, floret count per spikelet, and tiller count (*P*<0.001; **Table 3**). However, panicle form was not significantly associated with panicle length, hundred-seed weight, and seed area. Further analyses of multiple comparisons confirmed which specific panicle types were statistically different from one another for a given quantitative agronomic trait (**Supplementary Table S1**). For example, significant statistical differences were observed between very compact and compact, very compact and loose, and very compact and very loose panicle forms for fresh weight productivity per plant. For bioenergy production, compact panicles were statistically different from very compact, loose, and semi-loose forms for dry biomass productivity and very compact panicles were statistically different from compact and very loose forms for straw yield. For seed yield, very loose, loose, and semi-loose panicle forms were statistically significantly different for the compact forms, whereas very loose panicles were statistically significantly different from the semi-loose and very compact forms. A summary of descriptive statistics for the 11 quantitative agronomic traits is presented in **Supplementary Table S2**. Clearly, panicle morphology is a key and easily scorable morphological trait that is linked to other quantitative agronomic traits, which could be leveraged easily in future breeding programs.

**Table 3:** Analysis of variance (ANOVA) for the quantitative agronomic traits affected by panicle morphology.

No.	Traits	df	Sum of Squares	Mean Squares	F value	P value
1	Fresh weight	4	28177	7044	5.281	0.0004
2	Dry weight	4	5625	1406	4.89	0.0007
3	Seed yield	4	801	200	14.53	<0.0001
4	Straw yield	4	4104	1026	5.09	0.0005
5	Harvest index	4	0.1935	0.04838	27.07	<0.0001
6	Plant height	4	24468	6117	8.245	<0.0001
7	Panicle length	4	989.8	247.5	2.223	0.0661
8	Floret count	4	53.05	13.26	19.77	<0.0001
9	Tiller count	4	564.7	141.2	14.32	<0.0001
10	Hundred-seed weight	4	73.17	18.29	0.8597	0.4883
11	Seed area	4	0.01699	0.004247	0.715	0.5821

ANOVA was used investigate if panicle morphology showed a significant effect on the accessions, expressed as changes for 11 quantitative agronomic traits. All the measurements were taken on a single-plant basis. df = degrees of freedom. Significance was declared at *P*<0.0001.

### 3.3 Seed color

**Figure 4:**
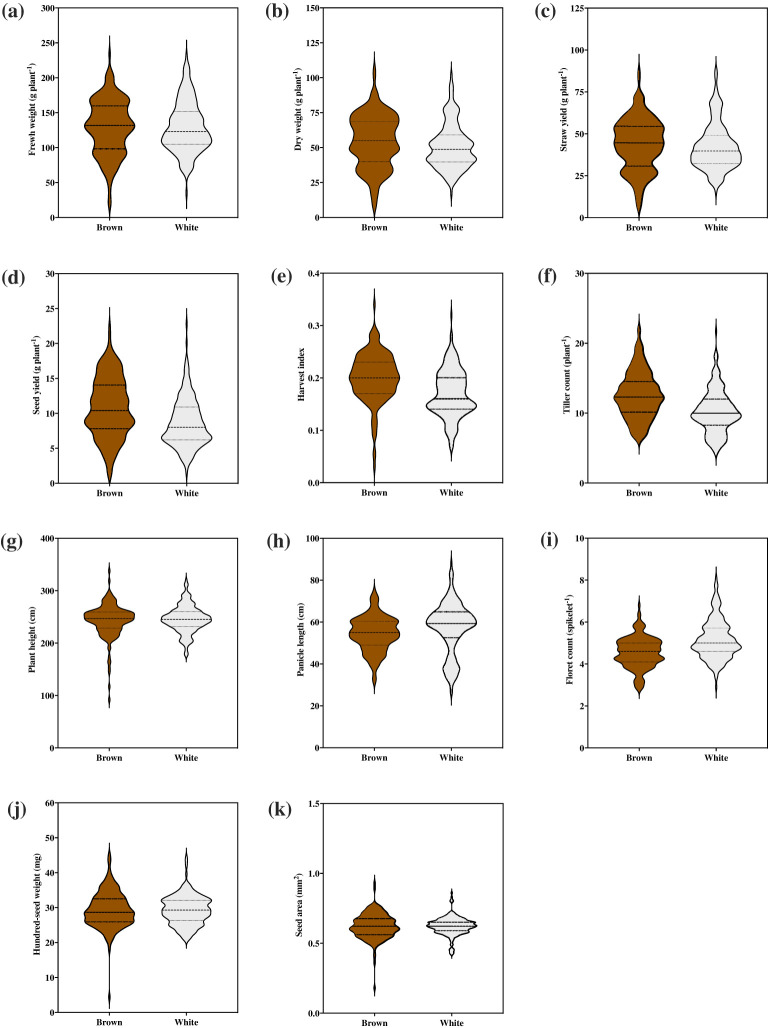
Association of seed color with 11 quantitative agronomic traits among 368 *E. tef* accessions. **(a)** fresh weight of biomass, **(b)** dry weight of biomass, **(c)** straw yield, **(d)** seed yield, **(e)**, harvest index, **(f)** tiller count, **(g)** plant height, **(h)** panicle length, **(i)** floret count, **(j)** hundred-seed weight, and **(k)** seed area. Violin plots show the distribution and density of the traits within each data set. Wider sections of the violin plots represent a greater frequency of accession with a particular value. The center dashed line represents median of the distribution whereas the fine dotted lines represent the lower (first) and upper (third) quartile of each distribution, respectively.

Two broad *E. tef* grain seed color categories were identified, namely brown and white (or ivory). Among the accessions, 203 (or 55%) were white seeded, whereas 165 (or 45%) were brown seeded (**Figure 2c, d**). Despite using single seeds when planting each accession, variation in seed color was observed in 13% of replicate plantings suggesting that some accessions were likely admixtures of two accessions. To address this variation, the consensus of the three replicates was used to score seed color (**Supplementary Table S8**). Violin plots were used to visualize differences in *E. tef* accessions across the 11 quantitative agronomic traits based upon seed color. Brown-seeded accessions showed higher median fresh and dry weight, straw yield, seed yield, harvest index, and tiller count compared with white-seeded accessions (**Figure 4**). In contrast, white-seeded accessions showed longer panicles and higher floret counts per panicle. The density plot graphs showed that seed color was associated with the 11 agronomic traits characterized in this study (**Supplementary Figure S2**). Notably, brown-seeded *E. tef* accessions showed higher straw yield per plant, higher seed yield, higher harvest index, and higher tiller count compared with white-seeded accessions. In contrast, white-seeded accessions showed longer panicles, higher hundred-seed weights, and higher seed areas (**Supplementary Figure S2**). Statistical analysis (*t*-test) showed that seed color was associated with significant differences among the 11 quantitative agronomic traits examined. Notably, seed yield, harvest index, panicle length, floret count per spikelet, and tiller count were statistically significantly different between the accessions based upon seed color (**Table 4**). Regarding panicle morphology, white-seeded accessions more often exhibited very compact, compact, and semi-loose panicle morphologies (**Figure 2e**). In contrast, accessions with brown-colored seeds more often exhibited loose and very loose panicle morphologies.

**Table 4:** Summary table for t-test analysis of seed color of 368 *E. tef* accessions for 11 quantitative agronomic traits.

Traits	Brown	White	P value	Remark
Fresh weight (g plant^-1^)	128.9	129.2	0.9271	ns
Dry weight (g plant^-1^)	54.4	51.0	0.0653	ns
Seed yield (g plant^-1^)	10.8	8.6	<0.0001	****
Straw yield (g plant^-1^)	43.6	42.3	0.3907	ns
Harvest index	0.20	0.17	<0.0001	****
Plant height (cm)	242.9	245.6	0.3715	ns
Panicle length (cm)	54.5	57.3	0.0099	**
Floret count (spikelet^-1^)	4.5	5.2	<0.0001	****
Tiller count (plant^-1^)	12.6	10.2	<0.0001	****
Hundred-seed weight (mg)	29.4	29.3	0.8897	ns
Seed area (mm^2^)	0.62	0.62	0.9197	ns

Significance was declared at *P*<0.0001 (****); P<0.01 (**); ns, not significant.

### 3.4 Biomass productivity

Fresh weight ranged from 3.6 grams plant^-1^ to 393.3 grams plant^-1^ (**Table 1**). Dry weight varied from 3.6 to 162.9 grams plant^-1^. Straw yield ranged from 1.7 grams plant^-1^ to 138.8 grams plant^-1^ (**Table 1**). Fresh weight, dry weight, and straw yield showed high genotypic coefficients of variance of 21.9, 25.6, and 26.6, respectively (**Table 2**). Likewise, the phenotypic coefficient of variance was high for all biomass productivity traits. Fresh weight, dry weight, and straw yield showed high phenotypic coefficients of variance of 39.5, 44.1, and 45.1, respectively (**Table 2**). ANOVA revealed that fresh weight, dry weight, and straw yield were associated with differences among accessions (*P*< 0.001, **Table 1**) and panicle morphology (P<0.001, **Table 3**). However, seed color showed no significant association with the three biomass productivity traits (**Table 4**).

### 3.5 Seed yield and harvest index

ANOVA showed that seed yield productivity was influenced by accessions or genotype (P< 0.05, **Table 1**), panicle form (P< 0.0001, **Table 3**), and seed color (*P<* 0.0001, **Table 4**). *E. tef* accessions exhibited great diversity in their seed yield ranging from 0.5–43.9 g plant^-1^ (**Table 1**). Seed yield showed high phenotypic and genotypic coefficients of variance of 56.2 and 31.1, respectively (**Table 2**). Heritability and genetic advance values for seed yield were in the moderate range.

Harvest index, as revealed by ANOVA and *t*-test, was associated with differences among accessions, (**Table 1**), panicle form (*P<*0.0001, **Table 3**), and seed color (*P<*0.0001, **Table 4**). The observed harvest indices ranged from 0.02 to 0.67 (**Table 1**). Harvest index showed moderate heritability, low genetic advance, and high genotypic coefficient of variance, phenotypic coefficient of variance, and genetic advance as a percent of mean (**Table 2**). The phenotypic and genotypic coefficients of variance for harvest index were 35.1 and 20, respectively. Harvest index showed a moderate negative correlation with plant height (r = -0.29) and straw yield (r = -0.24), whereas the association with seed yield (r = 0.43) was positive (**Supplementary Table S3**).

### 3.6 Plant height and tiller count

Plant height varied from 78.4 cm to 365.8 cm and showed the lowest coefficient of variance (9.3) among the agronomic traits analyzed (**Table 1**). The observed plant heights were taller than those typically observed in trials grown under field conditions likely due to the relatively reduced lighting conditions and close spacing conditions used. ANOVA showed that plant height was associated with differences among accessions (**Table 1**) and panicle form (*P<* 0.0001, **Table 3**). Plant height showed a positive association with seed area (r = 0.34), hundred-seed weight (r = 0.48), floret count (r = 0.24), panicle length (r = 0.55), fresh weight (r = 0.49), dry weight (r= 0.48), and straw yield (r = 0.53) (**Supplementary Table S3**). However, plant height showed no association with seed color (**Table 4**). Negative associations of plant height with tiller count (r=-0.29) and harvest index (r= -0.29) were observed (**Supplementary Table S3**). Phenotypic and genotypic coefficients of variance for plant height were 13.9 and 10.3, respectively (**Table 3**). ANOVA revealed the existence of a highly statistically significant difference among the number of tillers per plant (**Table 1**). Furthermore, tiller count was associated with differences among panicle form (*P*<0.0001, **Table 3**) and seed color (*P<* 0.0001, **Table 4**). Among the accessions, the minimum, maximum, and mean tiller counts plant^-1^ were 1, 32, and 11.3, respectively (**Table 1**). The coefficient of variance for tiller count was 31.1 (**Table 1**). Tiller count showed high phenotypic and genotypic coefficients of variance of 39.0 and 23.0, respectively, but only moderate heritability (36.7) (**Table 2**). Tiller count was negatively associated with floret count (r = -0.19) and panicle length (r = -0.34) (**Supplementary Table S3**). In contrast to plant height, tiller count did show an association with seed color (**Table 4**). Furthermore, tiller count was positively associated with seed yield (r = 0.43), fresh weight (r = 0.29), dry weight (r = 0.36), and straw yield (r = 0.31) (**Supplementary Table S3**).

### 3.7 Panicle length and floret count

Panicle length varied from 10.2 cm to 104.1 cm (**Table 1**). Panicle length associated with differences among accessions (*P<* 0.0001, **Table 1**) and seed color (*P<* 0.001, **Table 4**). However, ANOVA revealed that panicle length was not affected by panicle forms (*P* = 0.0661, **Table 3**). The minimum, maximum, and mean number of florets per spikelet were 2, 11, and 5, respectively (**Table 1**). Panicle length showed positive correlations with plant height (*r* = 0.55), hundred-seed weight (*r* = 0.44), floret count (*r* = 0.23), seed area (*r* = 0.24), biomass fresh weight (*r* = 0.24), and biomass dry weight (*r* = 0.21). The association of panicle length with seed yield (*r* = 0.12) was weakly positive (**Supplementary Table S3**). In contrast, panicle length was negatively correlated with tiller count (*r* = -0.34) (**Supplementary Table S3**). Panicle length was weakly correlated with seed yield and was not associated with any panicle form (**Table 3**). Moderate genotypic coefficients of variances of 16.4 and 13.1 were estimated for panicle length and floret count, respectively. However, high genotypic coefficients of variance of 23.3 and 25.9 were estimated for panicle length and floret count, respectively (**Table 2**).

### 3.8 Seed size and hundred-seed weight

ANOVA indicated that seed area (size) was associated with differences among accessions (P< 0.05, **Table 1**). In contrast, seed size was not affected prominently by panicle morphology (*P* = 0.5821, **Table 3**) and seed color (*P* = 0.9197, **Table 4**). Seed area varied from 0.13 to 1.36 mm^2^ (**Table 1**). Pearson correlation coefficient analysis indicated that seed size (area) was positively associated with panicle length (*r* = 0.24), plant height (*r* = 0.34), fresh weight (*r* = 0.17), dry weight (*r* = 0.15), and straw yield (*r* = 0.17) (**Supplementary Table S3**). Harvest index and seed area showed a significant but weak negative (*r* = -0.16) association (**Supplementary Table S3**). Seed area showed moderate phenotypic coefficients of variance and heritability estimates of 17.1 and 22.6, respectively (**Table 2**).

### 3.9 Correlation analysis

**Figure 5:**
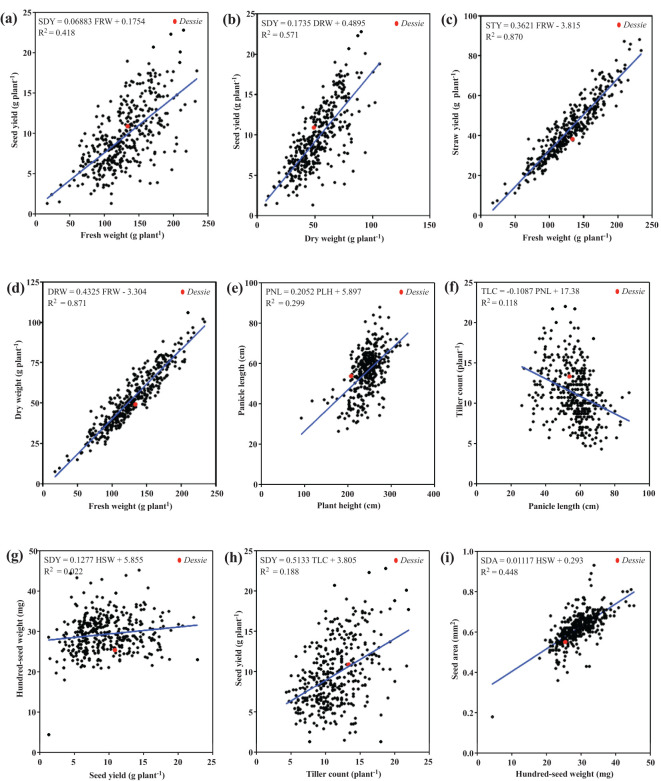
Correlation scatter plots of 368 *E. tef* accessions based upon 11 quantitative agronomic traits. Linear regression analysis was performed to quantitatively express the relationship between each pair of parameters. **(a)** seed yield and fresh weight, **(b)** seed yield and dry weight, **(c)** straw yield and fresh weight, **(d)** dry weight and fresh weight, **(e)** panicle length and plant height, **(f)** tiller count and panicle length, **(g)** hundred-seed weight and seed yield, **(h)** seed yield and tiller count, and **(i)** seed area and hundred-seed weight. The control cultivar named ‘*Dessie*’ is indicated as a red dot.

A pair-wise Pearson coefficient of correlation analysis was conducted to quantitatively express the magnitude and direction of relationship or association among the parameters. Scatter plot analyses were also performed to visualize the extent and patterns of association for all 11 quantitative agronomic traits and to highlight the large genetic variability and varying degrees of association among these traits (**Figure 5**). Linear regression equations were formulated to quantitatively express the relationship between any two agronomic parameters (**Figure 5**). Seed yield showed a positive correlation with above-ground biomass, both fresh and dry weights, with coefficients of determination (R^2^) values of 0.42 and 0.57, respectively (**Figure 5a, b**). Likewise, seed yield showed a positive relationship with hundred-seed weight and tiller count (**Figure 5g, h**). Notably, many of the accessions outperformed the named control cultivar (‘*Dessie’*) for several traits evaluated in the study. Correlation analysis revealed that seed yield showed a strong and positive association with biomass traits including fresh weight (r = 0.65, P< 0.0001), dry weight (r = 0.76, *P*< 0.0001; **Supplementary Table S3**), and straw yield (r = 0.63, *P*< 0.0001; **Supplementary Table S3**).

For biomass parameters, fresh weight was positively correlated with straw yield and dry weight, with coefficients of determination (R^2^) values of 0.87 for both comparisons. These observations were consistent with the strong and positive correlation among biomass traits. For instance, fresh weight was highly correlated with dry weight and straw yield (r = 0.93, **Supplementary Table S3**). Most of the 11 quantitative agronomic parameters showed positive correlations; however, a few did not. For example, the correlations between tiller count-panicle length, hundred-seed weight, and floret count were negative (**Figure 5f**). In addition, harvest index was negatively correlated with panicle length, straw yield, fresh weight, seed area, and floret count (**Supplementary Table S3**).

**Figure 6:**
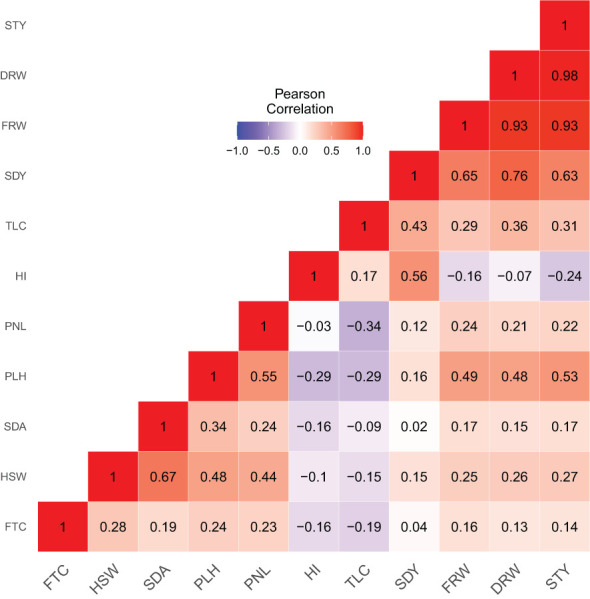
Correlation heatmap and Pearson correlation coefficients analysis of 368 *E. tef* accessions based upon 11 quantitative agronomic traits. Floret count per spikelet (FTC), hundred-seed weight (HSW), seed area (SDA), plant height (PLH), panicle length (PNL), harvest index (HI), tiller count (TLC), seed yield (SDY), fresh weight (FRW), dry weight (DRW), and straw yield (STY). Qualitative traits for panicle morphology and seed color were not included. Heat map scale indicates the degree of negative or positive correlations observed.

Pearson correlation analysis further revealed three categories of associations among the 11 quantitative agronomic traits (**Figure 6**; **Supplementary Table S3**): 1) strong to moderate positive association 2) moderate to weak negative association, and 3) no significant association. The relationship between straw yield and fresh weight, straw yield and dry weight, dry weight and seed yield, and dry weight and fresh weight were positive with Pearson correlation coefficients ranging from 0.76 to 0.98. The association of plant height with panicle length, hundred-seed weight, fresh weight, dry weight, and straw yield was positive and moderate with Pearson correlation coefficients ranging from 0.49 to 0.55. Similarly, hundred-seed weight was positively associated with seed yield and panicle length (*r* = 0.44 to 0.67). In addition, seed yield showed moderate to strong correlations with harvest index, fresh weight, and straw yield (*r* = 0.56 to 0.65). Floret count tended to show a positive but weak to moderate association with hundred-seed weight, seed area, plant height, fresh weight, dry weight, and straw yield. The associations of fresh weight, dry weight, and straw yield with floret count, hundred-seed weight, seed area, panicle length, and tiller count were positive but with weak to moderate associations. In contrast, the association of harvest index and tiller count with floret count, plant height, seed area, hundred-seed weight, and panicle length tended to be weakly negative. Harvest index also showed weak negative associations with fresh weight, dry weight, and straw yield.

### 3.10 Principal component analysis

Principal component analysis (PCA), a multivariate technique, was used to identify the main principal components, the contribution of each parameter to the total variance, and the relationship among the 11 quantitative agronomic traits (**Supplementary Tables S4**, **S5**). A scree plot, which is a plot connecting eigenvalues or percentage of explained variances of dimensions, was used to determine the number of principal components to keep for further analysis and inferences (**Supplementary Figure S3**). Accordingly, the first three principal components that accounted for about 72.6% of the total variation were maintained. The remaining variance was explained by the 4^th^ to 10^th^ principal components.

**Figure 7:**
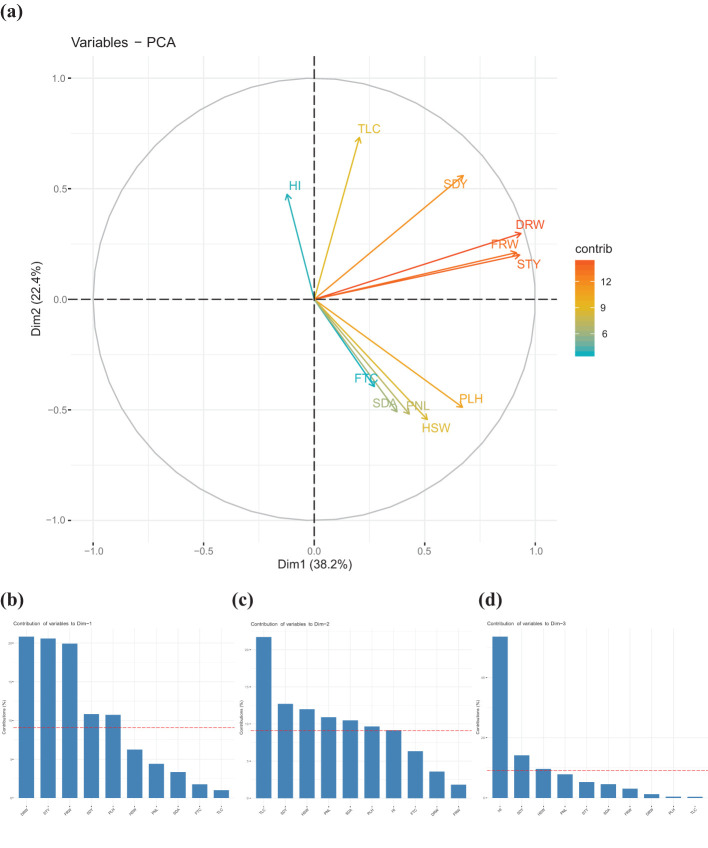
Principal component analysis of 368 teff accessions based on agronomic and morphological traits. **(a)** Magnitude of the relative contribution of each the agronomic parameters towards the total variance. Harvest index (HI), tiller count (TLC), seed yield (SDY), dry weight (DRW), fresh weight (FRW), straw yield (STY), plant height (PLH), hundred-seed weight (HSW), panicle length (PNL), seed area (SDA), and floret count per spikelet (FTC). Qualitative traits for panicle morphology and seed color were not included. Contribution of agronomic and morphological traits through the first three principal components. **(b)** contribution of agronomic traits through component 1, **(c)** contribution of agronomic traits through component 2, and **(d)** contribution of agronomic traits through component 3.

The biomass productivity traits contributed a significant portion of the variance through the first principal component. The relative contribution of each of the 11 quantitative agronomic parameters to the total variance and their relationship using the first two principal components were presented graphically to visualize overall variability (**Figure 7**). The angle between any two vectors indicates the direction and magnitude of association between the parameters. The lesser the angle between any two vectors the stronger their association. The PCA revealed that biomass productivity traits (*e.g.*, dry weight, fresh weight, and straw yield) contributed the most variance proportions followed closely by seed yield and that these parameters were highly correlated with each other. Although showing lower relative contributions, plant height, hundred-seed weight, panicle length, seed area, and floret count were correlated with each other.

The first principal component or dimension explained about 38.2% of the total variance that existed and included dry weight, straw yield, fresh weight, seed yield, and plant height (**Figure 7b**). The second principal component explained about 22.4% of the total variation and included tiller count, seed yield, hundred-seed weight, panicle length, seed area, plant height, and harvest index (**Figure 7c**). The third principal component explained about 12% of the variance and included harvest index, seed yield, and hundred-seed weight (**Figure 7d**).

### 3.11 Hierarchical cluster analysis

**Figure 8:**
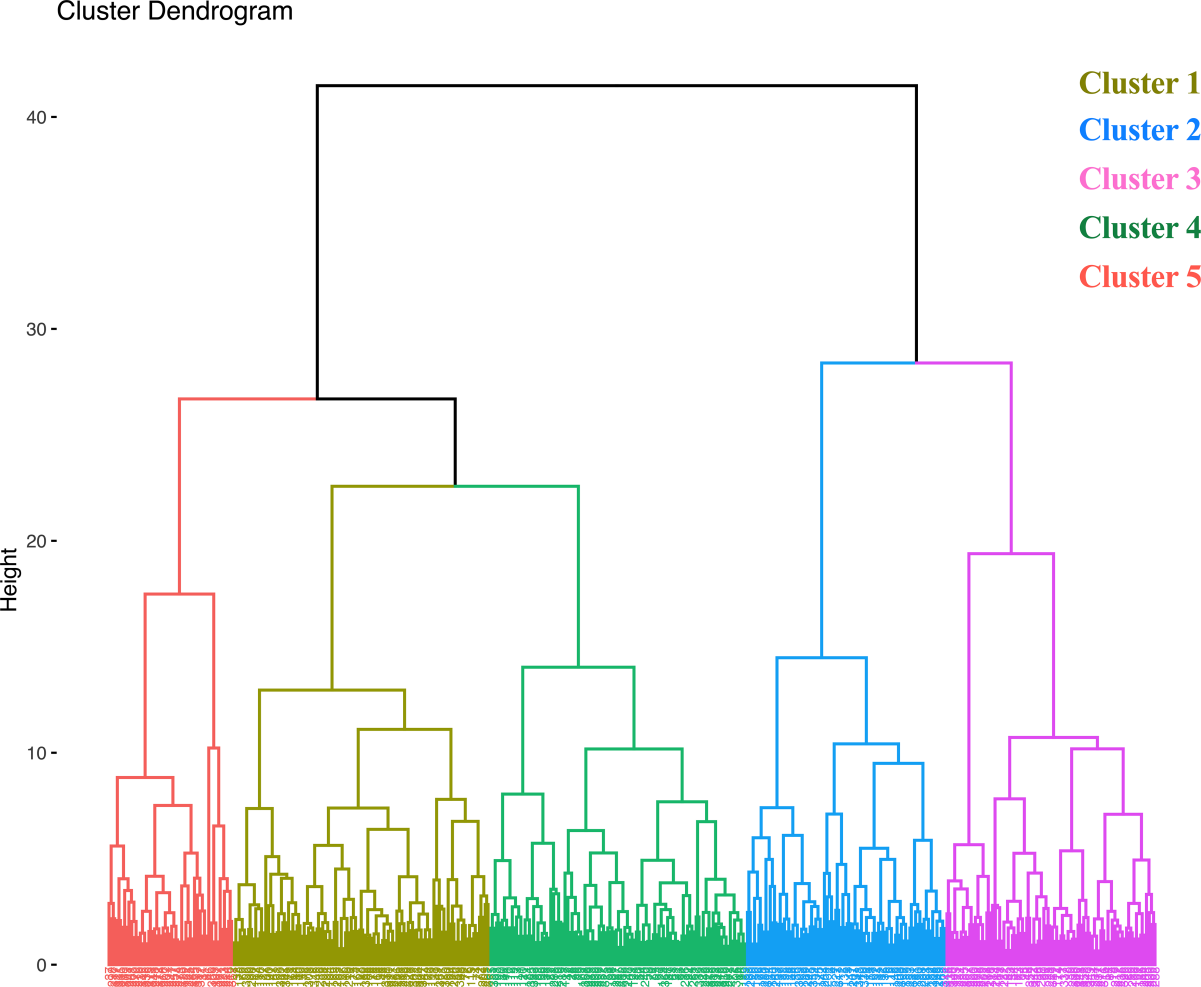
Dendrogram from hierarchical agglomerative clustering using 11 quantitative agronomic traits among 368 *E. tef* accessions. Data were subjected to normalization to avoid the effect of unit and magnitude difference among the different traits.

Hierarchical agglomerative clustering analysis was used to organize and identify which accessions shared key agronomic features based upon similarities among the 11 quantitative agronomic traits evaluated. Several methods including the elbow, silhouette, and gap statistics methods, were investigated and the elbow method was used to determine the optimum number of clusters within a data set (Maechler et al., 2013). The elbow method indicated five as the optimum number of clusters (**Supplementary Figure S4**). Clusters 1 and 4 each contained 90 accessions. Clusters 2 and 3 contained 70 and 74 accessions, respectively. Cluster 5 contained the lowest number of accessions (44) among the clusters (**Figure 8**). A summary of descriptive statistics for the five clusters is shown in **Supplementary Table S6**. Accessions within cluster 2 showed the highest mean fresh weight, dry weight, seed yield, and straw yield productivity, whereas cluster 4 and 5 showed the lowest values for these parameters. Violin plots of the five clusters are shown in **Supplementary Figure S5**. A heat map was also generated to visualize the existence of the inherent tendency of *E. tef* accessions to form clusters based upon the 11 quantitative agronomic traits (**Supplementary Figure S6**).

ANOVA showed that the five major cluster groups differed significantly from each other for all 11 quantitative agronomic traits (*P<* 0.0001; **Table 5**). Pair-wise comparisons of each cluster group revealed which clusters were different from the other. Accordingly, most cluster pair-wise comparisons yielded highly significant differences for biomass fresh weight and dry weight with the exception that no such significant difference in these traits was observed between clusters 1 and 3. Also, clusters 4 and 5 were similar in their dry biomass and straw yield. In addition, all clusters were significantly different from each other in terms of seed yield. Results of multiple comparisons for other parameters are shown in **Supplementary Table S7**.

**Table 5:** Analysis of variance (ANOVA) of the five clusters groups of 368 *E. tef* accessions.

No.	Traits	df	Sum of Squares	Mean Squares	F value	P value
1	Fresh weight	4	313680	78420	143.2	<0.0001
2	Dry weight	4	77072	19268	212.4	<0.0001
3	Seed yield	4	3448	861.9	132.8	<0.0001
4	Straw yield	4	49877	12469	165.2	<0.0001
5	Harvest index	4	0.1317	0.03293	16.82	<0.0001
6	Plant height	4	114882	28721	58.28	<0.0001
7	Panicle length	4	21187	5297	95.12	<0.0001
8	Floret count	4	1393	348.2	45.97	<0.0001
9	Tiller count	4	65.56	16.39	25.75	<0.0001
10	Hundred-seed weight	4	2724	680.9	48.72	<0.0001
11	Seed area	4	0.3529	0.08824	17.6	<0.0001

ANOVA revealed the existence of significant differences among the *E. tef* accessions (*n* = 368) for 11 quantitative agronomic traits following clustering into five major clusters. Summary of the ANOVA results presented for fresh weight, dry weight, seed yield, straw yield, harvest index, plant height, panicle length, floret count, tiller count, hundred-seed weight, and seed area. All the measurements were taken on single-plant basis. df = degrees of freedom. Significance was declared at *P*<0.0001.

#### 3.11.1 Cluster 1

Cluster 1 was one of the two largest clusters identified (90 accessions) and most accessions within this cluster exhibited mostly very compact or very loose panicles (**Supplementary Figure S7**) and a majority with brown seed (**Supplementary Figure S8**). Accessions within this cluster ranked second among all five clusters in terms of fresh weight, dry weight, seed yield, tiller count, and harvest index. ANOVA and multiple comparison of means (a mean separation procedure conducted following significant ANOVA results) showed that cluster 1 accessions were statistically significant from other accessions in some comparisons, but not others (**Supplementary Table S7**). For example, differences between adjacent clusters 1 and 4 in terms of plant height, seed area, and hundred-seed weight were non-significant. Cluster 1 was statistically equivalent to clusters 2 and 5 in terms of harvest index. Cluster 1 was also not significantly different from cluster 3 for fresh weight and dry weight. Furthermore, cluster 1 was not statistically different from clusters 2 and 5 for panicle length and tiller count, respectively. However, cluster 1 differed significantly from clusters 2 and 3 in terms of floret count but did not differ significantly from clusters 2 and 3 in other parameters.

#### 3.11.2 Cluster 2

Cluster 2 was the fourth largest cluster (70 accessions) identified and most accessions within this cluster exhibited mostly semi-loose, loose, and very loose panicles (**Supplementary Figure S7**) and a majority with brown seed (**Supplementary Figure S8**). Accessions within cluster 2 were notable in that they showed the highest agronomic performance in terms of fresh weight, dry weight, seed yield, straw yield, tiller count, and harvest index (**Supplementary Table S6**). Furthermore, cluster 2 accessions ranked second-highest among the clusters for floret count, hundred-seed weight, and seed area. A highly statistically significant difference was evident between cluster 2 and other clusters for fresh weight, dry weight, seed yield, straw yield, plant height, tiller count and hundred-seed weight (**Supplementary Table S7**). Furthermore, significant differences between cluster 2 and the other clusters were evident for harvest index, panicle length, floret count, and seed area with a few exceptions. For example, cluster 2 accessions showed no statistically significant difference between clusters 1 and 4 for harvest index and panicle length. Likewise, no differences were observed between cluster 2 and 4 for floret count per spikelet and seed area (**Supplementary Table S7**).

#### 3.11.3 Cluster 3

Cluster 3 was the third largest cluster (74 accessions) identified and most accessions within this cluster exhibited either very compact, compact, or semi-loose panicles (**Supplementary Figure S7**) and a majority with white seed (**Supplementary Figure S8**). *E. tef* accessions within cluster 3 showed the highest plant height, panicle length, floret count, hundred-seed weight, and seed area among the five clusters (**Supplementary Table S6**). In contrast, cluster 3 accessions showed the lowest harvest index and tiller count. Accession within cluster 3 ranked second in terms of fresh weight, dry weight, and straw yield. While accessions within cluster 3 showed significant differences from accessions within other cluster groups, pair-wise comparisons revealed no statistically significant differences between cluster 3 and cluster 1 for parameters such as fresh weight and dry weight. Likewise, accessions within cluster 3 showed no statistical difference with cluster 2, cluster 4, and cluster 5 for seed area, tiller count, and harvest index, respectively (**Supplementary Table S7**).

#### 3.11.4 Cluster 4

Cluster 4 was one of the two largest clusters identified (90 accessions) and accessions within this cluster exhibited all five panicle forms with semi-loose panicles being the most abundant (**Supplementary Figure S7**) and a majority with white seed (**Supplementary Figure S8**). Notably, cluster 4 accessions showed the lowest straw yield and tiller count plant^-1^ among the five clusters. Accessions within cluster 4 showed comparable plant height with accessions within clusters 1, 2, and 3. Accessions within cluster 4 also shared the highest harvest index (0.20) with clusters 1 and 2 and ranked second highest in panicle length (**Supplementary Table S6**). While accessions within cluster 4 showed significant differences from accessions within other cluster groups, pair-wise comparisons revealed no statistically significant differences between cluster 4 and 5 for parameters such as fresh weight, dry weight and straw yield. Furthermore, no significant differences were evident between cluster 4 with clusters 1 and 2 for harvest index, floret count, and seed area. Hundred-seed weight and plant height of accessions in cluster 4 were the same as those within cluster 1. Cluster 4 showed no significant difference with accessions within cluster 2 and 3 for panicle length and tiller count per spikelet, respectively (**Supplementary Table S7**).

#### 3.11.5 Cluster 5

Cluster 5 was the smallest (44 accessions) and accessions within this cluster exhibited mostly compact panicles, a lack of very compact panicles (**Supplementary Figure S7**), and a slight majority with white seed (**Supplementary Figure S8**). Cluster 5 was notable as it contained accessions with the lowest dry weight, seed yield, plant height, panicle length, floret count, seed area, and hundred-seed weight among the five clusters (**Supplementary Table S6**). The accessions within cluster 5 showed significant differences from accessions within other cluster groups in most cases. However, pair-wise comparisons revealed no statistically significant differences between other clusters in a few instances. For example, cluster 5 was not statistically different from cluster 4 in fresh weight, dry weight, and straw yield. Also, accessions within cluster 5 were comparable to those in cluster 3 for harvest index, and cluster 1 for floret count per spikelet (**Supplementary Table S7**). Overall, clustering analysis was extremely useful for categorizing accessions in terms of shared and contrasting agronomic traits with cluster 2 containing accessions with the highest biomass and seed production.

## 4 Discussion

Understanding existing genetic variation among *E. tef* accessions is a critical step towards ensuring the development of cultivars with more diverse genetic backgrounds, greater productivity, and enhanced resilience to the negative consequences of the changing climate. To investigate the potential genetic diversity of *E. tef* germplasm resources, we completed the first detailed phenotypic characterization of the national USDA-ARS *E. tef* germplasm collection available in the USA under optimum growth conditions. One accession (PI-337002) originated from Brazil and was determined to be *E. curvula*. An additional named cultivar called ‘*Dessie*’ was used as a control. From the evaluation of two qualitative and 11 quantitative agronomic traits we observed extensive diversity among the accessions for most of the traits examined, especially traits such as biomass yield, seed yield, plant height, and panicle length and to a lesser extent tiller count and hundred-seed weight (**Figure 1**). Such diversity could be leveraged through future selection and breeding efforts to improve selected traits such as grain and biomass yield, harvest index, and seed size or to select for accessions that show greater climate resiliency with reduced lodging or greater drought tolerance traits. Controlled environment studies are essential tools to control or manipulate factors of interest and provide valuable information about the performance of accessions under optimal growth conditions. However, for all traits evaluated here, we emphasize that replicated field trials are required to confirm results obtained under controlled conditions.

### 4.1 Panicle morphology, length, and floret count

A set of ~20 morphological, phenological, and agronomically important traits have been described for *E. tef* varieties (Assefa et al., 2011b). However, the morphology of the panicle or seed-bearing inflorescence, is one of the most important and readily scorable traits of *E. tef* that impacts seed yield and quality and is thus an important focus of crop improvement programs (Assefa et al., 2011b, 2017). Various classifications of *E. tef* morphology have been described. For example, five classes of panicle morphology including compact, semi-compact, semi-loose, loose, and very loose were defined (Ketema, 1997). Similarly, five types of panicles including very loose, loose, fairly loose, semi-compact, and compact were used to categorize panicle forms among 144 accessions from Ethiopia (Jifar et al., 2020). Likewise, five types of panicle morphologies including extra very loose (open), very loose (semi-open to semi-spreading), loose (spreading), semi-compact (semi-erect to spreading), and very compact (cylindrical with erect), were used to classify 2,084 accessions from Ethiopia within environmental contexts (Woldeyohannes et al., 2020).

In the current study, we identified five panicle forms as very loose, loose, semi-loose, compact, and very compact. Overall, we observed that 74% of panicles fell into the semi-loose, loose, or very loose categories with only 26% showing compact or very compact panicle forms (**Figure 2**). Our results were generally consistent with previous observations that a majority of *E. tef* accessions showed loose or fairly loose (75%) panicles compared with compact and semi-compact (22%) panicles (Ketema, 1997). Other surveys showed that open (95%) panicles were more common than closed (5%) panicles (Jifar et al., 2020) and that open or very loose and loose panicles were far more common than closed or semi-compact and very compact panicles (Woldeyohannes et al., 2020). Importantly, we observed that loose and very loose panicle forms showed the highest seed yields, harvest index, and tiller count plant^-1^ (**Figure 3**) and were more often associated with brown seed color. In contrast, very compact and compact panicles were more often associated with lower seed yield and white seeds (**Figure 2**). In contrast, *E. tef* accessions with very compact panicles showed the highest plant height and fresh and dry weight above-ground biomass productivity (**Figure 3**). Thus, future efforts to breed *E. tef* cultivars for improved grain production could focus on loose or very loose panicle forms, whereas efforts to breed for greater biomass for bioenergy production could focus on accessions with very compact panicle forms.

Like panicle morphology, the panicle length, is weakly associated with seed yield (**Figure 6**) and is also a major target of *E. tef* improvement programs. The range of panicle lengths observed in the current study performed under greenhouse conditions was far wider than those observed for limited numbers of accessions grown under field conditions, which ranged from 40–52 cm with a mean length of 45 cm (Teklu and Tefera, 2005). More recent surveys of *E. tef* panicle lengths reported ranges of 19.5–39.5 cm with a mean length of 30.4 cm (Jifar et al., 2020) and 21.6–42.1 cm with a mean length of 32.2 cm (Bayable et al., 2021). Larger surveys of 273 *E. tef* accessions grown in Israel reported larger variations in panicle length of 25–75 cm (Ben-Zeev et al., 2018). Panicle lengths varied the most in accessions with compact panicle forms (**Figure 3**).

Floret counts spikelet^-1^ varied from 2–11 with a mean of 5 among the accessions (**Figure 1**; **Table 1**). This number was similar to the mean number of kernels spikelet^-1^ of 6.8 reported for various varieties released in Ethiopia from 1960-1995 (Teklu and Tefera, 2005). Floret counts spikelet^-1^ were highest in the longest panicles of accessions with compact panicle forms and showed the highest range in variation among compact, semi-loose, loose, and very loose panicle forms (**Figure 3**). Floret counts spikelet^-1^ were highest in white-seeded accessions (**Figure 4**).

### 4.2 Seed color

Seed color is an important market value determinant for consumers and for market production decisions by *E. tef* grain producers. Ethiopian *E. tef* farmers will typically produce brown-seeded accessions for their own household consumption due to more desirable organoleptic qualities and greater nutritional value but prefer to sell the grain of white-seeded cultivars because of its higher market value. For example, Quncho, a popular, widely adapted, high-yielding improved cultivar released in 2006 was selected primarily due to its white seed color and reasonable tolerance to lodging (Assefa et al., 2011a).

Multiple classification systems for seed color have been developed for *E. tef*. Classification systems define multiple seed color classes including dark brown, reddish-brown, purple, medium brown, light tan, yellowish-white, and greyish-white, among other variations in seed color (Ebba, 1975; Assefa, 2003; Assefa et al., 2015; Merchuk-Ovnat et al., 2020). A comprehensive phenotypic characterization of 3,850 *E. tef* accessions from Ethiopia used the International Society Color Council-National Bureau of Standards system and the Royal Horticultural Society color chart system to arrive at five classes of seed color (*i.e.*, strong brown, light brown, medium brown to deep yellowish brown, yellowish white, and white) (Woldeyohannes et al., 2020). However, distinguishing among shades of brown and white colors can be challenging and so other researchers have typically used only 3-4 seed color classes (Jifar et al., 2020; Merchuk-Ovnat et al., 2020). The common denominator of these seed color classification systems is brown and white, so we chose to simplify our color classification system to group accessions according to these two classes. Based upon this simple scheme, we showed that white-seeded accessions were more frequent (55%) than brown-seeded accessions (45%) (**Figure 2**). Despite using single seeds to evaluate each accession, we noticed variations in seed color for 13% of accessions following replicate plantings suggesting that the current collection contained some accessions that lack genotypic uniformity. Future efforts to breed for specific shades of seed color would be desirable to not only expand the seed color palette for consumers, but also to help define the underlying genetic basis of seed color differences.

Interestingly, white-seeded accessions were more likely associated with very compact, compact, and semi-loose panicle forms, whereas brown-seeded accessions, were more likely associated with loose and very loose panicle forms (**Figure 3**). We also observed that white-seed accessions showed larger seeds as assessed by hundred-seed weight and seed area. However, the seed yield for white-seed accessions was lower than that of brown-seeded accessions, despite showing generally longer panicles and greater numbers of florets spikelet^-1^ (**Figure 4**). In contrast, brown-seeded *E. tef* accessions showed higher biomass production, seed yield, harvest index, and tiller count than white-seeded ones. Thus, future improvement efforts of *E. tef* accessions for seed production could focus on brown-seeded accessions with loose or very loose panicles for increasing overall seed yield. However, if larger seeds are desired, then white-seeded accessions might provide a better starting point for such breeding efforts.

### 4.3 Biomass productivity traits

As a C_4_ photosynthesis tropical grass species *E. tef* shows high biomass productivity and thus holds great potential as a dual-use bioenergy crop. To this end, we observed very high diversity in fresh weight, dry weight, and straw yields among the accessions evaluated (**Table 1**; **Figure 5**). These results were in general agreement with previous studies of *E. tef* accessions or recombinant inbred lines that showed a wide range of diversity for biomass productivity traits (Tefera et al., 2003; Chanyalew et al., 2009; Assefa et al., 2015). For example, previous studies have observed that fresh weight values ranged from 4.0–105 g plant^-1^ (Assefa et al., 2015). More recently, a survey of 317 accessions and three improved cultivars reported *E. tef* above ground straw yields of 16.1–23.1 g plant^-1^ under intensive field cultivation conditions (Bayable et al., 2021). Variations in biomass yield can vary depending upon day-length sensitivity of the accession with Ethiopian cultivars exhibiting a strong photoperiod response (van Delden et al., 2012). Although direct comparisons with field-grown plants and greenhouse-grown plants should not be made, our results showed a far wider range in variation of biomass productivity than previous field reports likely due to the large number of accessions tested and idealized growth conditions in the greenhouse. Nonetheless, our results highlight the enormous diversity present within the USDA-ARS collection and portend greater possible improvements in *E. tef* biomass productivity.

The diversity present within the accessions for biomass traits was best visualized through correlation analyses, which show the wide range of mean values for these traits (**Figure 5**). These results were similar to previous studies that showed that above-ground fresh and dry biomass were well correlated, but varied greatly among 196 recombinant inbred lines of *E. tef* (Chanyalew et al., 2009). Biomass traits were also well correlated with seed yield, plant height, and panicle length (**Figures 5**, **6**; **Supplementary Table S3**). Similar associations were observed previously between seed yield and above-ground biomass and plant height and panicle length (Tefera et al., 2003). The three biomass traits also contributed the most significant portion of variance to overall variance that was observed among the accessions and through the first principal component of traits (**Figure 7**). Based upon the large variation in biomass productivity and the number of accessions that showed greater biomass productivity than the ‘*Dessie*’ control line observed in the current study (**Figure 5**), we suggest that with additional selection, improvements in biomass production could be possible from existing accessions to foster the use of *E. tef* as a bioenergy feedstock. Under field conditions in Ethiopia, optimization of K fertilization rates (in the range of 60–90 kg ha^-1^) resulted in straw yields >8.7 Mg ha^-1^ depending upon field sites (Demiss et al., 2020). In Israel, above ground biomass productivity for hay or straw production for *E. tef* is estimated to be about 20 Mg ha^-1^ (Ben-Zeev et al., 2018). Total above-ground biomass production derived from multiple studies reported a range of 1.4–9.6 Mg ha^-1^ (Paff and Asseng, 2019). These biomass yields compare well with other bioenergy crops such as switchgrass, which shows biomass production in the range of 7-13 Mg ha^-1^ (Casler and Vogel, 2014; Casler, 2021).

Plant height is an important phenotypic trait linked with biomass production. We showed that plant height varied widely among the accessions (**Figure 1**) and was well correlated with panicle length, fresh weight, dry weight, straw yields, and hundred-seed weight and to a lesser extent with seed area, while being negatively correlated with harvest index (**Figure 6**; **Supplementary Table S3**). However, the height and the thin, hollow stems of *E. tef* accessions make them susceptible to stem or root lodging as the panicle develops and results in significant yield losses annually and reduced grain and straw quality (Ketema, 1993; Assefa et al., 2011b; Ben-Zeev et al., 2018; Paff and Asseng, 2019).

Under well-controlled greenhouse conditions, we observed plants that ranged from 78.7–365.8 cm and a mean of 244.4 cm in height (**Table 1**), which were generally taller than those obtained under field conditions probably due to reduced light intensities, which would be expected to trigger the shade response, resulting in taller plants. Under field conditions, plant heights have been reported with ranges of 20–156 cm (Assefa et al., 2015), 40-110 cm (Ben-Zeev et al., 2018), and 74–135 cm (Bayable et al., 2021). However, our results were higher than those from a report of greenhouse-grown *E. tef* accessions, which showed a plant height range of 147–200 cm (Merchuk-Ovnat et al., 2020). The greater height obtained in the current study likely arose from lower light conditions and longer day lengths due to the higher latitude of our location compared with Israel.

Selection of accessions with shorter stature and higher grain yield would be useful for developing more lodging-resistant cultivars (Jifar et al., 2017). One short-statured accession exhibited a minimal mean height of< 175 cm while maintaining good seed yields was identified (PI-494415) and would be a good candidate for future use or as a parental line for breeding programs to reduce lodging (**Supplementary Table S8**). However, this recommendation regarding plant stature is derived from plants grown under greenhouse conditions and such recommendations might not translate to performance under more highly variable, multi-environmental field conditions. Alternatively, short-statured cultivars can be isolated by the identification of mutants with reduced stature (Jöst et al., 2014) or through genome editing of the “green revolution” *SEMIDWARF 1* (*SD-1*) gene, which resulted in the creation of short-statured *E. tef* with reduced susceptibility to lodging at the heading stage compared with controls (Beyene et al., 2022).

In addition to plant height, tiller count or tiller capacity is an important quantitative agronomic trait that impacts overall productivity. Tiller are shoots that arise from the base of the plants derived from a single seed in important grain crops such as rice, wheat, and *E. tef.* Each tiller typically produces a seed-bearing inflorescence or panicle, thus contributing to both grain and biomass production. We observed that tiller count varied considerably from 1–32 with mean 11.3 (**Table 1**). Similar tiller counts ranging from 2-5 (Jifar et al., 2018), or 9-15 (Mihretie et al., 2021) were observed for plants grown under field conditions in Ethiopia. The two top accessions with the largest mean number of tillers (>18), while producing excellent above-ground biomass, included accessions PI-193511, and PI-193514, which produced a straw yield of 106.0 and 100.4 g plant^-1^, respectively. These two accessions would be good candidates for future use or as parental lines for breeding programs to increase biomass production (**Supplementary Table S8**). However, tiller count data derived from plants grown under greenhouse conditions might not translate to performance under more highly variable, multi-environmental field conditions.

### 4.4 Seed yield, harvest index, and seed size

The development of *E. tef* cultivars with high seed yield is a top priority for breeding efforts as improvements in edible grain food production has the highest economic impact for farmers (Assefa et al., 2011b). Our seed yield observations were generally consistent with previous studies, which showed wide variations in seed yield among different *E. tef* accessions (Assefa, 2003; Teklu and Tefera, 2005; Chanyalew et al., 2009; Assefa et al., 2015). However, variations in panicle development and growth can influence seed yield depending upon the day-length sensitivity of the cultivars under investigation (van Delden et al., 2012). More recently, a collection of 317 accessions and three improved cultivars revealed *E. tef* grain yields of 1.8–54.3 g plant^-1^ with conventional field management conditions, which rely solely upon ambient precipitation and inorganic fertilizer inputs, and 4.2–8.8 g plant^-1^ with intensive field cultivation conditions, which involved plant thinning and additional organic and inorganic fertilizer and irrigation inputs (Bayable et al., 2021). Depending upon the N fertilization rates used 46–115 N kg ha^-1^, *E. tef* grain yields can be improved and the amount needed can vary with the landscape (Gedamu et al., 2023). However, under the idealized conditions used in the current study, many accessions produced mean seed yields exceeding 10 g plant^-1^ illustrating the genetic potential for higher seed yields (**Figure 5**). The three top mean seed producers included accessions PI-494408, PI-494456, and PI-494369, which produced 22.8, 22.3, and 20.7 g plant^-1^, respectively, and thus would be good candidates for future use or as parental lines for breeding programs to increase seed yields (**Supplementary Table S8**). However, seed yield results derived from plants grown under greenhouse conditions might not translate to performance under more highly variable, multi-environmental field conditions.

Harvest index, the ratio of grain production to total plant biomass, serves as a useful measure of production efficiency in agriculture. Like grain production, harvest index varied widely among the accessions evaluated (**Table 1**). As might be expected, harvest index was positively correlated with seed yield (**Figure 6**; **Supplementary Table S3**), panicle form (**Table 3**), and seed color (**Table 4**). The current results were consistent with previous studies, which reported similar ranges of harvest indices that ranged from 0.05–0.39 plant^-1^ (Assefa et al., 2011b). A survey of 188 accessions reported a harvest index range of 0.15–0.24 plant^-1^ (Jifar et al., 2020). More recently, a collection of 317 accessions and three improved cultivars revealed *E. tef* harvest indices of 0.25–0.45 plant^-1^ with intensive field cultivation conditions (Bayable et al., 2021). As with seed yield, we observed a wider range of harvest indices with an upper limit of 0.67 plant^-1^ than field studies, again illustrating that the genetic potential for higher harvest indices exists among *E. tef* accessions. The two top mean harvest indices included accessions PI-494415 and PI-524445, which showed mean indices of 0.34 and 0.32, respectively. These accessions would be good candidates for future use or as parental lines for breeding programs to increase harvest indices (**Supplementary Table S8**). However, harvest index data derived from plants grown under greenhouse conditions might not translate to performance under more highly variable, multi-environmental field conditions.

Seed size, typically reported as hundred-seed or thousand seed weight, is often used as a key selection criterion for breeding efforts and as a measure of grain productivity. For example, ‘Quncho’ rapidly became a preferred cultivar in the major *E. tef* producing regions in Ethiopia due to its larger seed size (Belay et al., 2008; Assefa et al., 2011a). Hundred seed weight was weakly, but positively correlated with seed yield in the present study (**Figure 5g**; **Figure 6**). Other studies have reported a positive correlation of hundred-seed weight and overall grain yield as well (Belay et al., 2009; Chanyalew et al., 2009; Assefa et al., 2015).

In contrast to seed weight, seed area is typically not a parameter used for the evaluation of *E. tef* accessions because it can be difficult to measure due to the small size of *E. tef* seeds. *E. tef* seeds typically range in 0.9–1.7 mm in length and 0.7-1.0 mm in diameter (Ketema, 1997). Seed length was also reported to range from 1.0–1.3 mm (Merchuk-Ovnat et al., 2020). However, with imaging software and automated dimensional data capture, we were able to rapidly assess seed area, which was computed from seed width, length, and circularity (**Table 1**). As expected, seed area was highly correlated with hundred-seed weight (**Figure 5i**; **Figure 6**) as observed in previous studies (Belay et al., 2009).

### 4.5 Hierarchical cluster analysis

Multivariate techniques such as hierarchical cluster analysis have been used in various studies to organize *E. tef* accessions into groups, which can then be used to identify members within each group that share common phenotypes or members from groups with highly diverse phenotypes. For example, 144 heterogeneous *E. tef* accessions from ten major growing regions of Ethiopia were organized into eight distinct clusters based upon 18 quantitative agronomic traits to identify accessions for breeding efforts to improve grain yield (Adnew et al., 2005). Hierarchical clustering was also used to classify 15 landraces and three improved varieties of *E. tef* evaluated using 13 traits (Plaza-Wüthrich et al., 2013) and a collection of 36 brown-seeded *E. tef* accession phenotypes based upon 10 traits (Jifar et al., 2015). Cluster and divergence analysis grouped 28 selected *E. tef* progeny into six distinct clusters based upon 16 traits to identify semi-dwarf lines with higher grain yield (Jifar et al., 2017). Cluster analysis was also used to categorize 70 newly collected *E. tef* accessions into 12 clusters (Tsige et al., 2018). In another example, constellation plot clustering based upon seven phenotypic traits was used to organize 408 *E. tef* accessions from the Israel Gene Bank, to identify accessions with high phenotypic similarity, and to develop a diversity panel of 273 accessions (Ben-Zeev et al., 2018). This process helped streamline subsequent multi-year common garden field trials evaluating phenotypic diversity. Cluster analysis of 188 or 144 *E. tef* accessions from Ethiopia revealed six distinct clusters based upon five qualitative and seven quantitative traits (Jifar et al., 2018, 2020). Clustering based upon 12 administrative zones and four altitude zones revealed four and three clusters, respectively (Jifar et al., 2020). More recently, hierarchical clustering using the average linkage method was also employed to group 64 landraces into four clusters based upon 12 agronomic traits (Ashagrie et al., 2022).

Our hierarchical agglomerative clustering analysis of the accessions based upon 11 quantitative agronomic traits resulted in five distinct groups with shared characteristics (**Figure 8**; **Supplementary Table S6**). Tests for inherent tendency of clustering revealed that the optimum number of clusters was five (**Supplementary Figure S4**). ANOVA confirmed that the five clusters were highly statistically significantly (p<0.0001) different for all 11 quantitative agronomic traits (**Table 5**). The clustering analysis was extremely informative for organizing groups of accessions that shared key traits of agronomic interest. For example, cluster 2 contained accessions with the highest fresh and dry weight above-ground shoot biomass, straw yield, tiller count, seed yield, and plant height, whereas clusters 4 and 5 showed the lowest values for these quantitative traits (**Supplementary Figure S5**). Cluster 2 also contained the accessions with the highest hundred-seed weight and seed area. Interestingly, cluster 3 contained accessions with the highest plant height, panicle length, and floret counts. Clusters 3 and 5 held the accessions with the lowest harvest indices (**Supplementary Figure S5**). Pair-wise comparisons among the clusters revealed that most clusters were statistically distinct from one another based upon most parameters with some exceptions (**Supplementary Table S7**).

Distinct trends in qualitative traits among the clusters were also apparent. Cluster 1 contained the highest number of loose, very loose, and semi-loose panicle morphologies (**Supplementary Figure S7**), consistent with these panicle types exhibiting the greatest seed yields (**Figure 3**). In contrast, cluster 3 showed the highest number of very compact, compact, and semi-loose panicles, whereas cluster 1 contained the most very compact and very loose panicles (**Supplementary Figure S7**). Cluster 5 was comprised of accessions with the higher numbers of compact panicles relative to other classes of panicle forms. Seed color patterns were more evenly distributed among the different clusters with clusters 1 and 2 showing higher numbers of accessions with brown seeds, whereas clusters 3, 4, and 5, displayed the higher numbers of white-seeded accessions (**Supplementary Figure S8**). The resultant clusters organized the relatively heterogenous collection of accessions into homogeneous subsets to provide an opportunity to facilitate stratified sampling and a useful starting point for parental line selection.

## 5 Conclusions and future directions

This study provided the first detailed characterization of two qualitative traits and 11 quantitative traits of the national USDA-ARS *E. tef* germplasm collection under greenhouse conditions. Like previous field-based studies, our investigation revealed that the available germplasm collection from Ethiopia was highly diverse. The use of idealized growth conditions further revealed the existence of enormous genotypic potential for the development of cultivars with desirable agronomic traits such as improved biomass and seed yield production. Importantly, we observed that panicle morphology can play a major role in determining seed yield or biomass yield traits and provides a useful and easily scorable phenotyping tool for selection of these traits for future cultivar development. This study also revealed that white seeds were slightly larger than brown seeds, although this difference was not significant. Correlation analysis revealed that biomass traits (fresh weight, dry weight, and straw yield) and seed yield were well correlated overall and were the components most responsible for trait variance. Hierarchical clustering analysis of the accessions based upon their similarities for the 11 quantitative traits surveyed resulted in five distinct groups having shared phenotypes.

The accession recommendations made here are derived from plants grown under greenhouse conditions and that such recommendations should be validated under field conditions Thus, future field studies with these *E. tef* accessions will focus on the identification of accessions for the development of cultivars with maximal above-ground biomass for bioenergy production and enhanced resilience to the negative consequences of the changing climate including heat and drought stress tolerance. Additional studies will also focus on rapidly maturing accessions to develop cultivars with high seed yields to minimize seasonal or supplemental irrigation requirements. The selection of short-statured accessions with high seed yields will also be a high priority for the selection and development of lodging-resistant cultivars under field conditions. The extensive phenotypic data provided by this study also lays the foundation for future genome-wide association studies (GWAS) to map the relevant gene or genes associated with the quantitative and qualitative traits reported here.

## Data Availability

The original contributions presented in the study are included in the article/**Supplementary Material**. Further inquiries can be directed to the corresponding author.
