# Supplementary material for: Exploring the phenotypic diversity of Eragrostis tef for biomass and grain production under optimum growth conditions
Source: Front Plant Sci. 2025 Mar 17;16:1538510. doi: 10.3389/fpls.2025.1538510 (PMC11955633; doi:10.3389/fpls.2025.1538510)
Supplement: Supplementary file 1 [file DataSheet1.zip › Supplemental Figures S1-S8.DOCX]

**Supplemental Figures S1-S8**

**Figure S1. Density-plot distributions of quantitative traits of the 368 E. tef accessions associated with panicle morphology.** *E. tef* accessions were evaluated for a) fresh weight, b) dry weight, c) straw yield, d) seed yield, e) harvest index, f) hundred-seed weight, g) plant height, h) panicle length, i) seed area, j) tiller count, and k) floret count.

**Figure S2. Density-plot distributions of quantitative traits of the 368 E. tef accessions associated with seed color*.*** *E. tef* accessions were evaluated for a) fresh weight, b) dry weight, c) straw yield, d) seed yield, e) harvest index, f) hundred-seed weight, g) plant height, h) panicle length, i) seed area, j) tiller count, and k) floret count.

**Figure S3. Scree plot showing the percentage of variances explained by each principal component***.* Replication means computed for each accession were used in the principal component analysis (PCA). The first step in a PCA is to examine the proportion of total variance explained by individual components. Ten principal components were able to explain nearly 100% of the variation. Only the first three principal components which accounted about 72.6% of the variance were maintained for further analyses.

**Figure S4. Determination of the optimum number of clusters using the Elbow method of 368 *E. tef* accessions*.*** The method used to determine the optimum number of clusters based on a data set comprised of 11 quantitative agronomic traits for 368 *E. tef* accessions with three replications. Accordingly, five clusters were found to be optimal based upon the data set.

**Figure S5. Violin plots representing performance of E. tef accessions using quantitative agronomic traits under the five cluster groups*.*** The 368 *E. tef* accessions were clustered into five distinct groups based upon 11 quantitative agronomic traits. a) fresh weight, b) dry weight, c) straw yield, d) seed yield, e) harvest index, f) tiller count, g) plant height, h) panicle length, i) floret count, j) hundred-seed weight, and k) seed area. Violin plots show the distribution and density of the traits within each data set. Wider sections of the violin plots represent a greater frequency of accession with a particular value. The center dashed line represents median of the distribution whereas the fine dotted lines represent the lower (first) and upper (third) quartile of each distribution, respectively.

**Figure S6. Heat map to examine an inherent tendency of clustering among the 368 E. tef accessions based upon 11 quantitative agronomic traits.** The heat map was generated after normalizing the data set consisting of 11 quantitative agronomic traits in three replications. Normalization was done to avoid the effect of magnitude and unit differences among the 11 quantitative agronomic traits. Legend indicates the values of the relative distance (Euclidean) between each pair of rows and columns or between each pair of accessions.

**Figure S7. Panicle form distribution across five clusters of the 368 E. tef accessions.** Eleven quantitative agronomic traits were used for the hierarchical clustering analysis. Five cluster groups and five panicle forms were identified among the 368 *E. tef* accessions.


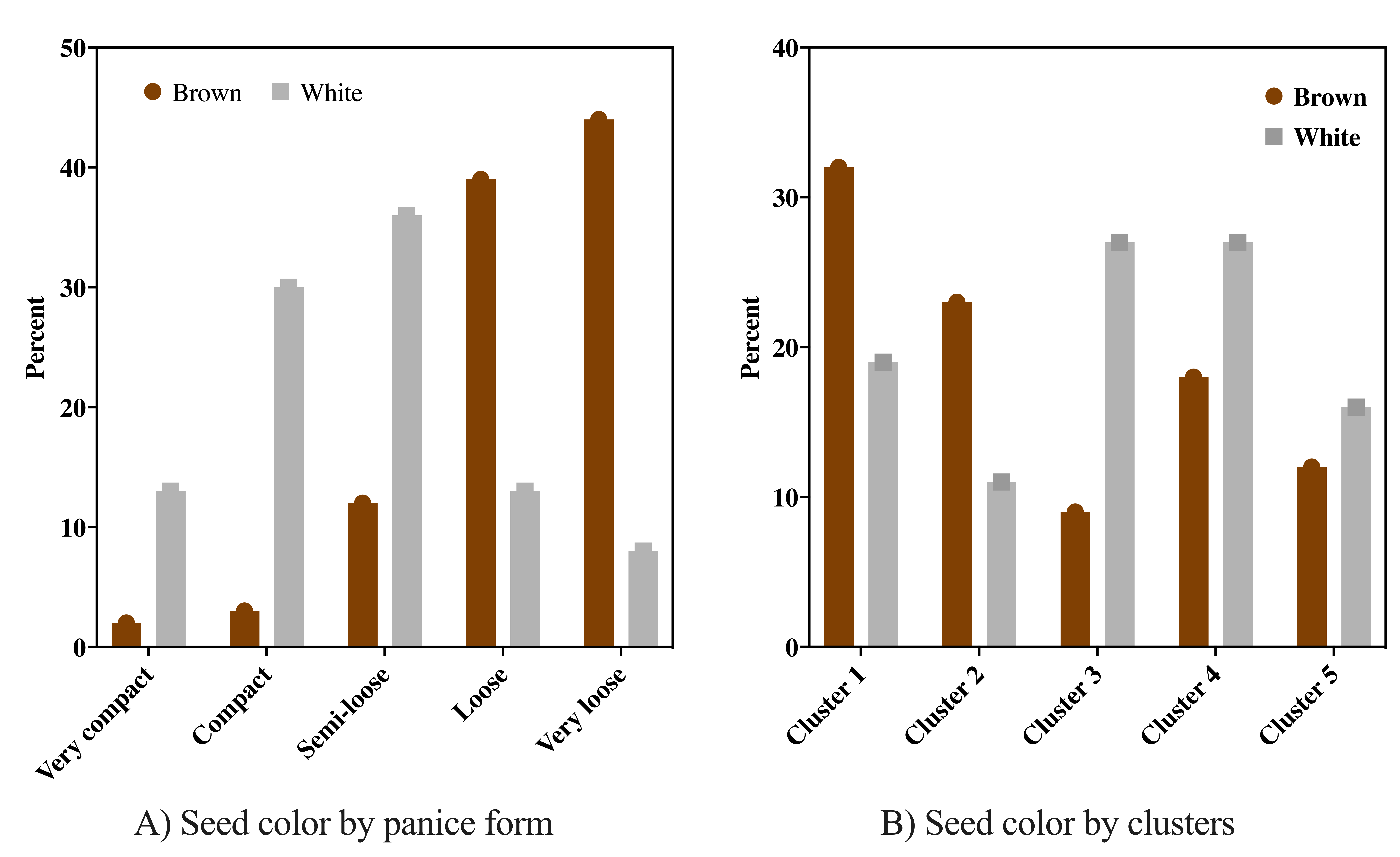


**Figure S8.** **The relationship between seed color among five hierarchical cluster groups***.* The percentage of accessions of each seed color class (brown or white) is show within each cluster.
