# Supplementary material for: Exploring the phenotypic diversity of Eragrostis tef for biomass and grain production under optimum growth conditions
Source: Front Plant Sci. 2025 Mar 17;16:1538510. doi: 10.3389/fpls.2025.1538510 (PMC11955633; doi:10.3389/fpls.2025.1538510)
Supplement: Supplementary file 1 [file DataSheet1.zip › Supplemental Tables S1-S8.DOCX]

**Supplemental Tables S1-S8**

**Table S1. Pair-wise comparison of effects of panicle forms on 11 quantitative agronomic traits of 368 *E. tef* accessions to investigate the statistical significance of panicle forms.** The traits considered were a) fresh weight, b) dry weight, c) seed yield, d) straw yield, e) harvest index, f) plant height, g) panicle length, h) tiller count, i) floret count, j) hundred-seed weight, k) seed area. The difference between any two pair of panicle means was declared at *P*<0.0001 (****); P<0.001 (***); P<0.01 (**); P<0.1 (*); ns, not significant. SE, standard error.

| 1. **Fresh weight (g plant^-1^)** | | | | | | |
| --- | --- | --- | --- | --- | --- | --- |
| **No.** | **Comparisons** | **Mean1** | **Mean2** | **SE of diff.** | **Summary** | **Adj. P value** |
| 1 | Very compact vs. Compact | 152.1 | 116.2 | 8.061 | *** | 0.0001 |
| 2 | Very compact vs. Semi loose | 152.1 | 132.0 | 7.669 | ns | 0.0688 |
| 3 | Very compact vs. Loose | 152.1 | 130.3 | 7.679 | * | 0.0387 |
| 4 | Very compact vs. Very loose | 152.1 | 126.3 | 7.722 | ** | 0.0082 |
| 5 | Compact vs. Semi loose | 116.2 | 132.0 | 5.905 | ns | 0.0611 |
| 6 | Compact vs. Loose | 116.2 | 130.3 | 5.918 | ns | 0.124 |
| 7 | Compact vs. Very loose | 116.2 | 126.3 | 5.973 | ns | 0.4446 |
| 8 | Semi loose vs. Loose | 132.0 | 130.3 | 5.371 | ns | 0.9979 |
| 9 | Semi loose vs. Very loose | 132.0 | 126.3 | 5.432 | ns | 0.8341 |
| 10 | Loose vs. Very loose | 130.3 | 126.3 | 5.446 | ns | 0.9482 |

| 1. **Dry weight (g plant^-1^)** | | | | | | |
| --- | --- | --- | --- | --- | --- | --- |
| **No.** | **Comparisons** | **Mean1** | **Mean2** | **SE of diff.** | **Summary** | **Adj. P value** |
| 1 | Very compact vs. Compact | 60.2 | 45.2 | 3.743 | *** | 0.0007 |
| 2 | Very compact vs. Semi loose | 60.2 | 53.2 | 3.56 | ns | 0.2774 |
| 3 | Very compact vs. Loose | 60.2 | 54.4 | 3.565 | ns | 0.4755 |
| 4 | Very compact vs. Very loose | 60.2 | 52.7 | 3.585 | ns | 0.2211 |
| 5 | Compact vs. Semi loose | 45.2 | 53.2 | 2.741 | * | 0.0316 |
| 6 | Compact vs. Loose | 45.2 | 54.4 | 2.748 | ** | 0.0081 |
| 7 | Compact vs. Very loose | 45.2 | 52.7 | 2.773 | ns | 0.0562 |
| 8 | Semi loose vs. Loose | 53.2 | 54.4 | 2.493 | ns | 0.9883 |
| 9 | Semi loose vs. Very loose | 53.2 | 52.7 | 2.522 | ns | 0.9997 |
| 10 | Loose vs. Very loose | 54.4 | 52.7 | 2.528 | ns | 0.9617 |

| 1. **Seed (g plant^-1^)** | | | | | | |
| --- | --- | --- | --- | --- | --- | --- |
| **No.** | **Comparisons** | **Mean1** | **Mean2** | **SE of diff.** | **Summary** | **Adj. P value** |
| 1 | Very compact vs. Compact | 8.9 | 6.8 | 0.8193 | ns | 0.0949 |
| 2 | Very compact vs. Semi loose | 8.9 | 9.4 | 0.7794 | ns | 0.9593 |
| 3 | Very compact vs. Loose | 8.9 | 10.5 | 0.7805 | ns | 0.2147 |
| 4 | Very compact vs. Very loose | 8.9 | 11.1 | 0.7848 | * | 0.035 |
| 5 | Compact vs. Semi loose | 6.8 | 9.4 | 0.6001 | *** | 0.0002 |
| 6 | Compact vs. Loose | 6.8 | 10.5 | 0.6015 | **** | <0.0001 |
| 7 | Compact vs. Very loose | 6.8 | 11.1 | 0.6071 | **** | <0.0001 |
| 8 | Semi loose vs. Loose | 9.4 | 10.5 | 0.5459 | ns | 0.2457 |
| 9 | Semi loose vs. Very loose | 9.4 | 11.1 | 0.5521 | * | 0.0171 |
| 10 | Loose vs. Very loose | 10.5 | 11.1 | 0.5535 | ns | 0.8147 |

Table S1 (continued…)

| 1. **Straw yield (g plant^-1^)** | | | | | | |
| --- | --- | --- | --- | --- | --- | --- |
| **No.** | **Comparisons** | **Mean1** | **Mean2** | **SE of diff.** | **Summary** | **Adj. P value** |
| 1 | Very compact vs. Compact | 51.8 | 38.2 | 3.134 | *** | 0.0002 |
| 2 | Very compact vs. Semi loose | 51.8 | 43.7 | 2.981 | ns | 0.0577 |
| 3 | Very compact vs. Loose | 51.8 | 43.9 | 2.985 | ns | 0.0658 |
| 4 | Very compact vs. Very loose | 51.8 | 41.6 | 3.002 | ** | 0.0069 |
| 5 | Compact vs. Semi loose | 38.2 | 43.7 | 2.295 | ns | 0.1121 |
| 6 | Compact vs. Loose | 38.2 | 43.9 | 2.3 | ns | 0.0984 |
| 7 | Compact vs. Very loose | 38.2 | 41.6 | 2.322 | ns | 0.5873 |
| 8 | Semi loose vs. Loose | 43.7 | 43.9 | 2.088 | ns | >0.9999 |
| 9 | Semi loose vs. Very loose | 43.7 | 41.6 | 2.111 | ns | 0.8448 |
| 10 | Loose vs. Very loose | 43.9 | 41.6 | 2.117 | ns | 0.8137 |

| 1. **Harvest index** | | | | | | |
| --- | --- | --- | --- | --- | --- | --- |
| **No.** | **Comparisons** | **Mean1** | **Mean2** | **SE of diff.** | **Summary** | **Adj. P value** |
| 1 | Very compact vs. Compact | 0.16 | 0.15 | 0.009331 | ns | 0.9485 |
| 2 | Very compact vs. Semi loose | 0.16 | 0.18 | 0.008876 | ns | 0.1632 |
| 3 | Very compact vs. Loose | 0.16 | 0.19 | 0.008888 | *** | 0.0005 |
| 4 | Very compact vs. Very loose | 0.16 | 0.22 | 0.008938 | **** | <0.0001 |
| 5 | Compact vs. Semi loose | 0.15 | 0.18 | 0.006835 | *** | 0.001 |
| 6 | Compact vs. Loose | 0.15 | 0.19 | 0.00685 | **** | <0.0001 |
| 7 | Compact vs. Very loose | 0.15 | 0.22 | 0.006914 | **** | <0.0001 |
| 8 | Semi loose vs. Loose | 0.18 | 0.19 | 0.006216 | ns | 0.0662 |
| 9 | Semi loose vs. Very loose | 0.18 | 0.22 | 0.006287 | **** | <0.0001 |
| 10 | Loose vs. Very loose | 0.19 | 0.22 | 0.006304 | ** | 0.006 |

| 1. **Plant height (cm)** | | | | | | |
| --- | --- | --- | --- | --- | --- | --- |
| **No.** | **Comparisons** | **Mean1** | **Mean2** | **SE of diff.** | **Summary** | **Adj. P value** |
| 1 | Very compact vs. Compact | 266.6 | 241.7 | 6.012 | *** | 0.0004 |
| 2 | Very compact vs. Semi loose | 266.6 | 245.0 | 5.719 | ** | 0.0018 |
| 3 | Very compact vs. Loose | 266.6 | 247.6 | 5.727 | ** | 0.0093 |
| 4 | Very compact vs. Very loose | 266.6 | 234.7 | 5.758 | **** | <0.0001 |
| 5 | Compact vs. Semi loose | 241.7 | 245.0 | 4.404 | ns | 0.9475 |
| 6 | Compact vs. Loose | 241.7 | 247.6 | 4.413 | ns | 0.6673 |
| 7 | Compact vs. Very loose | 241.7 | 234.7 | 4.455 | ns | 0.5105 |
| 8 | Semi loose vs. Loose | 245.0 | 247.6 | 4.005 | ns | 0.964 |
| 9 | Semi loose vs. Very loose | 245.0 | 234.7 | 4.051 | ns | 0.084 |
| 10 | Loose vs. Very loose | 247.6 | 234.7 | 4.061 | * | 0.0134 |

| 1. **Panicle length (cm)** | | | | | | |
| --- | --- | --- | --- | --- | --- | --- |
| **No.** | **Comparisons** | **Mean1** | **Mean2** | **SE of diff.** | **Summary** | **Adj. P value** |
| 1 | Very compact vs. Compact | 60.2 | 54.5 | 2.329 | ns | 0.1068 |
| 2 | Very compact vs. Semi loose | 60.2 | 57.0 | 2.215 | ns | 0.6025 |
| 3 | Very compact vs. Loose | 60.2 | 56.3 | 2.218 | ns | 0.3966 |
| 4 | Very compact vs. Very loose | 60.2 | 54.4 | 2.231 | ns | 0.0756 |
| 5 | Compact vs. Semi loose | 54.5 | 57.0 | 1.706 | ns | 0.5868 |
| 6 | Compact vs. Loose | 54.5 | 56.3 | 1.71 | ns | 0.8377 |
| 7 | Compact vs. Very loose | 54.5 | 54.4 | 1.726 | ns | >0.9999 |
| 8 | Semi loose vs. Loose | 57.0 | 56.3 | 1.551 | ns | 0.9903 |
| 9 | Semi loose vs. Very loose | 57.0 | 54.4 | 1.569 | ns | 0.4727 |
| 10 | Loose vs. Very loose | 56.3 | 54.4 | 1.573 | ns | 0.7649 |

Table S1 (continued…)

| 1. **Tiller count (plant^-1^)** | | | | | | |
| --- | --- | --- | --- | --- | --- | --- |
| **No.** | **Comparisons** | **Mean1** | **Mean2** | **SE of diff.** | **Summary** | **Adj. P value** |
| 1 | Very compact vs. Compact | 9.1 | 10.2 | 0.6929 | ns | 0.4903 |
| 2 | Very compact vs. Semi loose | 9.1 | 10.7 | 0.6592 | ns | 0.1197 |
| 3 | Very compact vs. Loose | 9.1 | 11.6 | 0.66 | ** | 0.0023 |
| 4 | Very compact vs. Very loose | 9.1 | 13.2 | 0.6637 | **** | <0.0001 |
| 5 | Compact vs. Semi loose | 10.2 | 10.7 | 0.5076 | ns | 0.8947 |
| 6 | Compact vs. Loose | 10.2 | 11.6 | 0.5087 | ns | 0.0717 |
| 7 | Compact vs. Very loose | 10.2 | 13.2 | 0.5135 | **** | <0.0001 |
| 8 | Semi loose vs. Loose | 10.7 | 11.6 | 0.4616 | ns | 0.3333 |
| 9 | Semi loose vs. Very loose | 10.7 | 13.2 | 0.4669 | **** | <0.0001 |
| 10 | Loose vs. Very loose | 11.6 | 13.2 | 0.4681 | ** | 0.0058 |

| 1. **Floret count (spikelet^-1^)** | | | | | | |
| --- | --- | --- | --- | --- | --- | --- |
| **No.** | **Comparisons** | **Mean1** | **Mean2** | **SE of diff.** | **Summary** | **Adj. P value** |
| 1 | Very compact vs. Compact | 4.9 | 5.5 | 0.1808 | ** | 0.0082 |
| 2 | Very compact vs. Semi loose | 4.9 | 5.1 | 0.172 | ns | 0.5226 |
| 3 | Very compact vs. Loose | 4.9 | 4.7 | 0.1722 | ns | 0.8467 |
| 4 | Very compact vs. Very loose | 4.9 | 4.4 | 0.1732 | * | 0.0493 |
| 5 | Compact vs. Semi loose | 5.5 | 5.1 | 0.1324 | ns | 0.0861 |
| 6 | Compact vs. Loose | 5.5 | 4.7 | 0.1327 | **** | <0.0001 |
| 7 | Compact vs. Very loose | 5.5 | 4.4 | 0.134 | **** | <0.0001 |
| 8 | Semi loose vs. Loose | 5.1 | 4.7 | 0.1204 | ** | 0.0024 |
| 9 | Semi loose vs. Very loose | 5.1 | 4.4 | 0.1218 | **** | <0.0001 |
| 10 | Loose vs. Very loose | 4.7 | 4.4 | 0.1221 | ns | 0.1026 |

| 1. **Hundred-seed weight (plant^-1^)** | | | | | | |
| --- | --- | --- | --- | --- | --- | --- |
| **No.** | **Comparisons** | **Mean1** | **Mean2** | **SE of diff.** | **Summary** | **Adj. P value** |
| 1 | Very compact vs. Compact | 28.0 | 29.3 | 1.018 | ns | 0.6771 |
| 2 | Very compact vs. Semi loose | 28.0 | 29.5 | 0.9686 | ns | 0.5272 |
| 3 | Very compact vs. Loose | 28.0 | 29.7 | 0.9698 | ns | 0.3689 |
| 4 | Very compact vs. Very loose | 28.0 | 29.2 | 0.9752 | ns | 0.7088 |
| 5 | Compact vs. Semi loose | 29.3 | 29.5 | 0.7458 | ns | 0.9995 |
| 6 | Compact vs. Loose | 29.3 | 29.7 | 0.7474 | ns | 0.9822 |
| 7 | Compact vs. Very loose | 29.3 | 29.2 | 0.7544 | ns | >0.9999 |
| 8 | Semi loose vs. Loose | 29.5 | 29.7 | 0.6783 | ns | 0.9961 |
| 9 | Semi loose vs. Very loose | 29.5 | 29.2 | 0.686 | ns | 0.9952 |
| 10 | Loose vs. Very loose | 29.7 | 29.2 | 0.6878 | ns | 0.9444 |

| 1. **Seed area (mm^2^)** | | | | | | |
| --- | --- | --- | --- | --- | --- | --- |
| **No.** | **Comparisons** | **Mean1** | **Mean2** | **SE of diff.** | **Summary** | **Adj. P value** |
| 1 | Very compact vs. Compact | 0.62 | 0.63 | 0.01701 | ns | 0.9994 |
| 2 | Very compact vs. Semi loose | 0.62 | 0.61 | 0.01618 | ns | 0.9562 |
| 3 | Very compact vs. Loose | 0.62 | 0.63 | 0.0162 | ns | 0.9942 |
| 4 | Very compact vs. Very loose | 0.62 | 0.62 | 0.01629 | ns | >0.9999 |
| 5 | Compact vs. Semi loose | 0.63 | 0.61 | 0.01246 | ns | 0.739 |
| 6 | Compact vs. Loose | 0.63 | 0.63 | 0.01249 | ns | 0.9995 |
| 7 | Compact vs. Very loose | 0.63 | 0.62 | 0.0126 | ns | 0.9879 |
| 8 | Semi loose vs. Loose | 0.61 | 0.63 | 0.01133 | ns | 0.5093 |
| 9 | Semi loose vs. Very loose | 0.61 | 0.62 | 0.01146 | ns | 0.9349 |
| 10 | Loose vs. Very loose | 0.63 | 0.62 | 0.01149 | ns | 0.9368 |

**Table S2. Descriptive statistics of agronomic traits of *E. tef* associated with panicle morphology.** Descriptive statistics of the 11 quantitative agronomic traits by panicle morphology was summarized as a) fresh weight, b) dry weight, c) seed yield, d) straw yield, e) harvest index, f) plant height, g) panicle length, h) tiller count, i) floret count, j) hundred-seed weight, k) seed area. *n* = 368.

| **No.** | **a. Fresh weight**  **(g plant^-1^)** | **Panicle forms** | | | | |
| --- | --- | --- | --- | --- | --- | --- |
|  |  | **Very compact** | **Compact** | **Semi-loose** | **Loose** | **Very loose** |
| 1 | #Accessions | 30 | 65 | 93 | 92 | 88 |
| 2 | Minimum | 90.7 | 35.2 | 68.7 | 17.6 | 24.1 |
| 3 | 25% Percentile | 119.4 | 97.4 | 105.4 | 107.5 | 92.25 |
| 4 | Median | 156 | 110.4 | 131.4 | 132.3 | 128.3 |
| 5 | 75% Percentile | 174.4 | 131.3 | 159.2 | 159.3 | 154.2 |
| 6 | Maximum | 218.2 | 231.4 | 216.7 | 233.8 | 209.8 |
| 7 | Mean | 152.1 | 116.2 | 132 | 130.3 | 126.3 |
| 8 | Std. Deviation | 35.56 | 33.72 | 34.72 | 36.37 | 40.61 |
| 9 | Std. Error of Mean | 6.492 | 4.183 | 3.601 | 3.792 | 4.329 |
| 10 | Lower 95% CI | 138.8 | 107.9 | 124.8 | 122.8 | 117.7 |
| 11 | Upper 95% CI | 165.4 | 124.6 | 139.1 | 137.8 | 134.9 |

| **No.** | **b. Dry weight**  **(g plant^-1^)** | **Panicle forms** | | | | |
| --- | --- | --- | --- | --- | --- | --- |
|  |  | **Very compact** | **Compact** | **Semi-loose** | **Loose** | **Very loose** |
| 1 | #Accessions | 30 | 65 | 93 | 92 | 88 |
| 2 | Minimum | 33.4 | 17.2 | 25.9 | 7.5 | 9.7 |
| 3 | 25% Percentile | 46.78 | 35.55 | 40 | 41.6 | 36.9 |
| 4 | Median | 59.45 | 42.8 | 51.3 | 53.95 | 53.85 |
| 5 | 75% Percentile | 75.75 | 52.3 | 65.05 | 68.15 | 66.28 |
| 6 | Maximum | 93.6 | 102.1 | 96.8 | 100.4 | 106 |
| 7 | Mean | 60.21 | 45.19 | 53.16 | 54.38 | 52.67 |
| 8 | Std. Deviation | 16.79 | 14.07 | 15.93 | 17.81 | 18.98 |
| 9 | Std. Error of Mean | 3.065 | 1.745 | 1.651 | 1.857 | 2.023 |
| 10 | Lower 95% CI | 53.94 | 41.7 | 49.88 | 50.69 | 48.65 |
| 11 | Upper 95% CI | 66.48 | 48.68 | 56.44 | 58.07 | 56.69 |

| **No.** | **c. Seed yield**  **(g plant^-1^)** | **Panicle forms** | | | | |
| --- | --- | --- | --- | --- | --- | --- |
|  |  | **Very compact** | **Compact** | **Semi-loose** | **Loose** | **Very loose** |
| 1 | #Accessions | 30 | 65 | 93 | 92 | 88 |
| 2 | Minimum | 5.3 | 1.5 | 1.3 | 1.3 | 2.4 |
| 3 | 25% Percentile | 6.85 | 5.05 | 6.5 | 7.4 | 8.5 |
| 4 | Median | 8.55 | 6.6 | 8.9 | 10.2 | 11.35 |
| 5 | 75% Percentile | 9.9 | 8.15 | 11.45 | 13.43 | 14.15 |
| 6 | Maximum | 16.6 | 14.7 | 22.8 | 20.7 | 22.3 |
| 7 | Mean | 8.873 | 6.832 | 9.409 | 10.53 | 11.13 |
| 8 | Std. Deviation | 2.51 | 2.628 | 3.868 | 4.119 | 4.092 |
| 9 | Std. Error of Mean | 0.4582 | 0.3259 | 0.4011 | 0.4295 | 0.4362 |
| 10 | Lower 95% CI | 7.936 | 6.181 | 8.612 | 9.673 | 10.26 |
| 11 | Upper 95% CI | 9.81 | 7.483 | 10.21 | 11.38 | 11.99 |

Table S2 (Continued…)

| **No.** | **d. Straw yield**  **(g plant^-1^)** | **Panicle forms** | | | | |
| --- | --- | --- | --- | --- | --- | --- |
|  |  | **Very compact** | **Compact** | **Semi-loose** | **Loose** | **Very loose** |
| 1 | #Accessions | 30 | 65 | 93 | 92 | 88 |
| 2 | Minimum | 28.3 | 15.7 | 21.1 | 6.2 | 7.3 |
| 3 | 25% Percentile | 37.58 | 30.7 | 32.85 | 35.08 | 28.33 |
| 4 | Median | 48.9 | 36.6 | 42.3 | 43.5 | 42.5 |
| 5 | 75% Percentile | 68.33 | 43.4 | 52.9 | 53.63 | 52.75 |
| 6 | Maximum | 85.8 | 88.1 | 83.2 | 82.6 | 87.2 |
| 7 | Mean | 51.75 | 38.18 | 43.73 | 43.87 | 41.57 |
| 8 | Std. Deviation | 15.99 | 11.9 | 13.23 | 14.63 | 15.58 |
| 9 | Std. Error of Mean | 2.919 | 1.477 | 1.372 | 1.525 | 1.66 |
| 10 | Lower 95% CI | 45.78 | 35.23 | 41.01 | 40.84 | 38.27 |
| 11 | Upper 95% CI | 57.72 | 41.13 | 46.46 | 46.9 | 44.87 |

| **No.** | **e. Harvest index (ratio)** | **Panicle forms** | | | | |
| --- | --- | --- | --- | --- | --- | --- |
|  |  | **Very compact** | **Compact** | **Semi-loose** | **Loose** | **Very loose** |
| 1 | #Accessions | 30 | 65 | 93 | 92 | 88 |
| 2 | Minimum | 0.07 | 0.07 | 0.03 | 0.05 | 0.09 |
| 3 | 25% Percentile | 0.1075 | 0.13 | 0.15 | 0.17 | 0.19 |
| 4 | Median | 0.16 | 0.15 | 0.18 | 0.19 | 0.21 |
| 5 | 75% Percentile | 0.2025 | 0.16 | 0.21 | 0.22 | 0.25 |
| 6 | Maximum | 0.25 | 0.22 | 0.28 | 0.29 | 0.34 |
| 7 | Mean | 0.157 | 0.1502 | 0.177 | 0.1934 | 0.215 |
| 8 | Std. Deviation | 0.05107 | 0.0331 | 0.04646 | 0.03915 | 0.04355 |
| 9 | Std. Error of Mean | 0.009324 | 0.004105 | 0.004817 | 0.004081 | 0.004642 |
| 10 | Lower 95% CI | 0.1379 | 0.142 | 0.1674 | 0.1853 | 0.2058 |
| 11 | Upper 95% CI | 0.1761 | 0.1584 | 0.1866 | 0.2015 | 0.2242 |

| **No.** | **f. Plant height (cm)** | **Panicle forms** | | | | |
| --- | --- | --- | --- | --- | --- | --- |
|  |  | **Very compact** | **Compact** | **Semi-loose** | **Loose** | **Very loose** |
| 1 | #Accessions | 30 | 65 | 93 | 92 | 88 |
| 2 | Minimum | 211.5 | 177.6 | 196.9 | 91.9 | 116.8 |
| 3 | 25% Percentile | 241 | 229.9 | 232 | 240.6 | 218.8 |
| 4 | Median | 260.8 | 244.1 | 244.5 | 250.7 | 238.8 |
| 5 | 75% Percentile | 291.8 | 258.5 | 257.4 | 261.3 | 254.8 |
| 6 | Maximum | 320 | 293.4 | 318.6 | 337.8 | 297.6 |
| 7 | Mean | 266.6 | 241.7 | 245 | 247.6 | 234.7 |
| 8 | Std. Deviation | 30.25 | 26.59 | 21.41 | 28.75 | 30.35 |
| 9 | Std. Error of Mean | 5.523 | 3.298 | 2.22 | 2.998 | 3.235 |
| 10 | Lower 95% CI | 255.3 | 235.1 | 240.6 | 241.7 | 228.3 |
| 11 | Upper 95% CI | 277.8 | 248.3 | 249.4 | 253.6 | 241.1 |

Table S2 (Continued …)

| **No.** | **g. Panicle length (cm)** | **Panicle forms** | | | | |
| --- | --- | --- | --- | --- | --- | --- |
|  |  | **Very compact** | **Compact** | **Semi-loose** | **Loose** | **Very loose** |
| 1 | #Accessions | 30 | 65 | 93 | 92 | 88 |
| 2 | Minimum | 37.3 | 26.2 | 33.9 | 32.8 | 30.5 |
| 3 | 25% Percentile | 56.23 | 38 | 50.1 | 51.65 | 47.93 |
| 4 | Median | 59.8 | 58.6 | 58 | 56.4 | 55.8 |
| 5 | 75% Percentile | 65.65 | 64.55 | 64.55 | 61.4 | 60.5 |
| 6 | Maximum | 72.2 | 88.1 | 84.2 | 83.2 | 72.4 |
| 7 | Mean | 60.2 | 54.51 | 57.01 | 56.28 | 54.43 |
| 8 | Std. Deviation | 7.746 | 15.34 | 10.8 | 8.18 | 8.837 |
| 9 | Std. Error of Mean | 1.414 | 1.903 | 1.12 | 0.8529 | 0.942 |
| 10 | Lower 95% CI | 57.3 | 50.71 | 54.78 | 54.59 | 52.56 |
| 11 | Upper 95% CI | 63.09 | 58.31 | 59.23 | 57.98 | 56.31 |

| **No.** | **h. Tiller count**  **(plant^-1^)** | **Panicle forms** | | | | |
| --- | --- | --- | --- | --- | --- | --- |
|  |  | **Very compact** | **Compact** | **Semi-loose** | **Loose** | **Very loose** |
| 1 | #Accessions | 30 | 65 | 93 | 92 | 88 |
| 2 | Minimum | 4.7 | 5 | 4.3 | 6 | 7 |
| 3 | 25% Percentile | 6.3 | 8.7 | 8 | 9.3 | 10.3 |
| 4 | Median | 8.7 | 9.7 | 10.3 | 11.3 | 12.7 |
| 5 | 75% Percentile | 11.48 | 11.3 | 12.5 | 13.23 | 15.7 |
| 6 | Maximum | 17.7 | 18 | 22 | 21.7 | 21.7 |
| 7 | Mean | 9.113 | 10.23 | 10.69 | 11.56 | 13.17 |
| 8 | Std. Deviation | 3.228 | 2.677 | 3.376 | 2.984 | 3.319 |
| 9 | Std. Error of Mean | 0.5894 | 0.332 | 0.3501 | 0.3111 | 0.3538 |
| 10 | Lower 95% CI | 7.908 | 9.567 | 9.995 | 10.94 | 12.46 |
| 11 | Upper 95% CI | 10.32 | 10.89 | 11.39 | 12.17 | 13.87 |

| **No.** | **i. Floret count**  **(spikelet^-1^)** | **Panicle forms** | | | | |
| --- | --- | --- | --- | --- | --- | --- |
|  |  | **Very compact** | **Compact** | **Semi-loose** | **Loose** | **Very loose** |
| 1 | #Accessions | 30 | 65 | 93 | 92 | 88 |
| 2 | Minimum | 3.8 | 3.8 | 3.3 | 2.9 | 2.8 |
| 3 | 25% Percentile | 4.375 | 4.8 | 4.6 | 4.2 | 3.9 |
| 4 | Median | 4.8 | 5.3 | 5.1 | 4.6 | 4.3 |
| 5 | 75% Percentile | 5 | 6.05 | 5.7 | 5.175 | 4.975 |
| 6 | Maximum | 5.9 | 8.1 | 7.7 | 7.8 | 7 |
| 7 | Mean | 4.87 | 5.474 | 5.139 | 4.695 | 4.394 |
| 8 | Std. Deviation | 0.5646 | 0.9848 | 0.816 | 0.7966 | 0.7822 |
| 9 | Std. Error of Mean | 0.1031 | 0.1221 | 0.08462 | 0.08305 | 0.08338 |
| 10 | Lower 95% CI | 4.659 | 5.23 | 4.971 | 4.53 | 4.229 |
| 11 | Upper 95% CI | 5.081 | 5.718 | 5.307 | 4.86 | 4.56 |

Table S2 (Continued …)

| **No.** | **j. Hundred-seed weight (plant^-1^)** | **Panicle forms** | | | | |
| --- | --- | --- | --- | --- | --- | --- |
|  |  | **Very compact** | **Compact** | **Semi-loose** | **Loose** | **Very loose** |
| 1 | #Accessions | 30 | 65 | 93 | 92 | 88 |
| 2 | Minimum | 21.2 | 21 | 20.9 | 4.4 | 18 |
| 3 | 25% Percentile | 26.13 | 24.95 | 26.8 | 26.2 | 26 |
| 4 | Median | 28.3 | 29.9 | 29.3 | 29.85 | 28.65 |
| 5 | 75% Percentile | 29.7 | 32.4 | 32.3 | 32.9 | 32.28 |
| 6 | Maximum | 32.1 | 42.9 | 44.4 | 43.3 | 45.2 |
| 7 | Mean | 27.97 | 29.32 | 29.48 | 29.73 | 29.21 |
| 8 | Std. Deviation | 2.34 | 4.625 | 4.202 | 5.262 | 4.857 |
| 9 | Std. Error of Mean | 0.4272 | 0.5737 | 0.4358 | 0.5486 | 0.5178 |
| 10 | Lower 95% CI | 27.1 | 28.17 | 28.61 | 28.64 | 28.18 |
| 11 | Upper 95% CI | 28.84 | 30.46 | 30.34 | 30.82 | 30.24 |

| **No.** | **k. Seed area (mm^2^)** | **Panicle forms** | | | | |
| --- | --- | --- | --- | --- | --- | --- |
|  |  | **Very compact** | **Compact** | **Semi-loose** | **Loose** | **Very loose** |
| 1 | #Accessions | 30 | 65 | 93 | 92 | 88 |
| 2 | Minimum | 0.5 | 0.43 | 0.42 | 0.18 | 0.36 |
| 3 | 25% Percentile | 0.6 | 0.59 | 0.575 | 0.5725 | 0.5725 |
| 4 | Median | 0.63 | 0.63 | 0.61 | 0.63 | 0.61 |
| 5 | 75% Percentile | 0.65 | 0.67 | 0.65 | 0.68 | 0.66 |
| 6 | Maximum | 0.68 | 0.75 | 0.93 | 0.89 | 0.77 |
| 7 | Mean | 0.6217 | 0.6255 | 0.6103 | 0.6283 | 0.6193 |
| 8 | Std. Deviation | 0.03922 | 0.06144 | 0.08011 | 0.0942 | 0.07362 |
| 9 | Std. Error of Mean | 0.007161 | 0.007621 | 0.008308 | 0.009821 | 0.007848 |
| 10 | Lower 95% CI | 0.607 | 0.6103 | 0.5938 | 0.6088 | 0.6037 |
| 11 | Upper 95% CI | 0.6363 | 0.6408 | 0.6268 | 0.6478 | 0.6349 |

**Table S3. Pearson correlation coefficients of 11 quantitative agronomic traits of the 368 *E. tef* accessions.** Pearson coefficients values range from -1 to +1, where -1 indicates perfect negative associations while +1 indicates perfect positive associations between two given agronomic parameters. P-values are given for each coefficient.

**Table S4. Eigen vectors, eigenvalues, and variance explained by principal components.** The first three principal components alone accounted for 72.6% of the total variation that existed among the 368 *E. tef* accessions based upon 11 quantitative agronomic traits used in the study. Dim, dimension.

|  | Eigenvalue | Variance | Cumulative variance |
| --- | --- | --- | --- |
| Dim 1 | 4.198419342 | 38.16744856 | 38.16745 |
| Dim 2 | 2.467098873 | 22.42817157 | 60.59562 |
| Dim 3 | 1.315079113 | 11.95526467 | 72.55088 |
| Dim 4 | 1.029775760 | 9.36159782 | 81.91248 |
| Dim 5 | 0.845022524 | 7.68202294 | 89.59451 |
| Dim 6 | 0.441828752 | 4.01662502 | 93.61113 |
| Dim 7 | 0.300576645 | 2.73251495 | 96.34365 |
| Dim 8 | 0.284333240 | 2.58484763 | 98.92849 |
| Dim 9 | 0.083390593 | 0.75809630 | 99.68659 |
| Dim 10 | 0.032964744 | 0.29967949 | 99.98627 |
| Dim 11 | 0.001510416 | 0.01373105 | 100.0000 |

**Table S5. Proportion of the variance contribution of 11 quantitative agronomic traits among the 368 *E. tef* accessions based upon through the first three principal component.** Dim, dimension.

| Trait | Dim1 | Dim2 | Dim3 |
| --- | --- | --- | --- |
| Fresh weight | 0.9144772 | 0.2111660 | -0.20008122 |
| Dry weight | 0.9351933 | 0.2977670 | -0.12683682 |
| Seed yield | 0.6742647 | 0.5600286 | 0. 43126494 |
| Straw yield | 0.9296528 | 0.2006559 | -0.26353233 |
| Harvest index | -0.1228929 | 0.4752075 | 0.83938805 |
| Plant height | 0.6710979 | -0.4881637 | -0.06941156 |
| Panicle length | 0.4299655 | -0.5187894 | 0.32099638 |
| Tiller count | 0.2046900 | 0.7321127 | -0.06400215 |
| Floret count | 0.2722753 | -0.3949035 | 0.02541890 |
| Hundred-seed weight | 0.5118796 | -0.5439088 | 0.35548526 |
| Seed area | 0.3745384 | -0.5088270 | 0.24490650 |

**Table S6. Descriptive statistics of agronomic and morphological traits of *E. tef* categorized by the five cluster groups.** Descriptive statistics of the 11 agronomic traits by cluster group was summarized as a) fresh weight, b) dry weight, c) seed yield, d) straw yield, e) harvest index, f) plant height, g) panicle length, h) tiller count, i) floret count, j) hundred-seed weight, k) seed area. n = 368.

| **No.** | **a. Fresh weight**  **(g plant^-1^)** | **Cluster groups** | | | | |
| --- | --- | --- | --- | --- | --- | --- |
|  |  | **1** | **2** | **3** | **4** | **5** |
| 1 | #Accessions | 90 | 70 | 74 | 90 | 44 |
| 2 | Minimum | 88.8 | 130.8 | 76.7 | 64.8 | 17.6 |
| 3 | 25% Percentile | 122.3 | 164.1 | 116.7 | 84.3 | 73.3 |
| 4 | Median | 133.4 | 174.1 | 138.1 | 94.4 | 98.1 |
| 5 | 75% Percentile | 144.7 | 186.6 | 161.4 | 107.8 | 113.7 |
| 6 | Maximum | 179.2 | 233.8 | 218.2 | 138.1 | 156.0 |
| 7 | Mean | 133.4 | 175.3 | 141.0 | 95.8 | 94.6 |
| 8 | Std. Deviation | 17.0 | 21.8 | 30.4 | 16.9 | 33.3 |
| 9 | Std. Error of Mean | 1.8 | 2.6 | 3.5 | 1.8 | 5.0 |
| 10 | Lower 95% CI | 129.9 | 170.1 | 133.9 | 92.3 | 84.5 |
| 11 | Upper 95% CI | 137.0 | 180.5 | 148.0 | 99.3 | 104.7 |
| 12 | Range | 90.4 | 103 | 141.5 | 73.3 | 138.4 |
| 13 | CV | 14.82 | 12.44 | 21.54 | 17.63 | 35.16 |

| **No.** | **b. Dry weight**  **(g plant^-1^)** | **Cluster groups** | | | | |
| --- | --- | --- | --- | --- | --- | --- |
|  |  | **1** | **2** | **3** | **4** | **5** |
| 1 | #Accessions | 90 | 70 | 74 | 90 | 44 |
| 2 | Minimum | 39.7 | 63.9 | 30.1 | 20.4 | 7.5 |
| 3 | 25% Percentile | 48.5 | 69.9 | 47.5 | 31.7 | 28.0 |
| 4 | Median | 54.3 | 74.8 | 53.8 | 36.7 | 35.1 |
| 5 | 75% Percentile | 59.7 | 79.4 | 64.2 | 41.4 | 42.4 |
| 6 | Maximum | 75.9 | 106.0 | 93.6 | 54.1 | 58.6 |
| 7 | Mean | 54.7 | 76.5 | 56.6 | 36.7 | 35.5 |
| 8 | Std. Deviation | 7.6 | 9.2 | 12.4 | 6.6 | 12.7 |
| 9 | Std. Error of Mean | 0.8 | 1.1 | 1.4 | 0.7 | 1.9 |
| 10 | Lower 95% CI | 53.1 | 74.3 | 53.7 | 35.3 | 31.6 |
| 11 | Upper 95% CI | 56.3 | 78.7 | 59.5 | 38.1 | 39.3 |
| 12 | Range | 30.6 | 42.1 | 63.5 | 33.7 | 51.1 |
| 13 | CV | 14 | 12.04 | 21.9 | 17.9 | 35.83 |

| **No.** | **c. Seed yield**  **(g plant^-1^)** | **Cluster groups** | | | | |
| --- | --- | --- | --- | --- | --- | --- |
|  |  | **1** | **2** | **3** | **4** | **5** |
| 1 | #Accessions | 90 | 70 | 74 | 90 | 44 |
| 2 | Minimum | 1.3 | 9.1 | 3.6 | 2.9 | 1.3 |
| 3 | 25% Percentile | 8.8 | 12.5 | 6.6 | 6.2 | 3.8 |
| 4 | Median | 11.0 | 15.0 | 8.3 | 6.9 | 5.5 |
| 5 | 75% Percentile | 12.8 | 16.8 | 10.0 | 8.6 | 6.6 |
| 6 | Maximum | 17.4 | 22.8 | 16.5 | 13.1 | 13.4 |
| 7 | Mean | 10.7 | 15.0 | 8.5 | 7.3 | 5.5 |
| 8 | Std. Deviation | 2.9 | 2.9 | 2.5 | 2.0 | 2.2 |
| 9 | Std. Error of Mean | 0.3 | 0.3 | 0.3 | 0.2 | 0.3 |
| 10 | Lower 95% CI | 10.1 | 14.3 | 7.9 | 6.9 | 4.8 |
| 11 | Upper 95% CI | 11.3 | 15.7 | 9.0 | 7.7 | 6.2 |
| 12 | Range | 11.7 | 13.7 | 12.9 | 10.2 | 12.1 |
| 13 | CV | 24.71 | 19.52 | 29.66 | 26.93 | 40.71 |

Table S6 (Continued…)

| **No.** | **d. Straw yield**  **(g plant^-1^)** | **Cluster groups** | | | | |
| --- | --- | --- | --- | --- | --- | --- |
|  |  | **1** | **2** | **3** | **4** | **5** |
| 1 | #Accessions | 90 | 70 | 74 | 90 | 44 |
| 2 | Minimum | 31.9 | 48.4 | 25.5 | 16.7 | 6.2 |
| 3 | 25% Percentile | 39.1 | 54.6 | 40.1 | 25.5 | 21.6 |
| 4 | Median | 43.1 | 59.9 | 44.9 | 29.4 | 30.5 |
| 5 | 75% Percentile | 48.8 | 65.7 | 52.7 | 33.4 | 36.3 |
| 6 | Maximum | 61.3 | 88.1 | 85.8 | 42.3 | 50.5 |
| 7 | Mean | 44.0 | 61.5 | 48.1 | 29.6 | 29.7 |
| 8 | Std. Deviation | 6.2 | 8.8 | 11.9 | 5.6 | 11.3 |
| 9 | Std. Error of Mean | 0.7 | 1.0 | 1.4 | 0.6 | 1.7 |
| 10 | Lower 95% CI | 42.7 | 59.4 | 45.4 | 28.4 | 26.3 |
| 11 | Upper 95% CI | 45.3 | 63.6 | 50.9 | 30.8 | 33.1 |
| 12 | Range | 30.7 | 39.7 | 60.3 | 25.6 | 44.3 |
| 13 | CV | 14.86 | 14.26 | 24.74 | 18.94 | 38 |

| **No.** | **e. Harvest index** | **Cluster groups** | | | | |
| --- | --- | --- | --- | --- | --- | --- |
|  |  | **1** | **2** | **3** | **4** | **5** |
| 1 | #Accessions | 90 | 70 | 74 | 90 | 44 |
| 2 | Minimum | 0.03 | 0.11 | 0.05 | 0.09 | 0.07 |
| 3 | 25% Percentile | 0.17 | 0.17 | 0.13 | 0.17 | 0.13 |
| 4 | Median | 0.20 | 0.20 | 0.16 | 0.20 | 0.15 |
| 5 | 75% Percentile | 0.23 | 0.22 | 0.18 | 0.23 | 0.19 |
| 6 | Maximum | 0.28 | 0.28 | 0.24 | 0.29 | 0.34 |
| 7 | Mean | 0.19 | 0.20 | 0.15 | 0.20 | 0.16 |
| 8 | Std. Deviation | 0.04 | 0.04 | 0.04 | 0.04 | 0.06 |
| 9 | Std. Error of Mean | 0.00 | 0.00 | 0.00 | 0.00 | 0.01 |
| 10 | Lower 95% CI | 0.19 | 0.19 | 0.14 | 0.19 | 0.14 |
| 11 | Upper 95% CI | 0.20 | 0.21 | 0.16 | 0.21 | 0.18 |
| 12 | Range | 0.25 | 0.17 | 0.19 | 0.2 | 0.27 |
| 13 | CV | 22.2 | 18.69 | 26.64 | 21.39 | 38.43 |

| **No.** | **f. Plant height (cm)** | **Cluster groups** | | | | |
| --- | --- | --- | --- | --- | --- | --- |
|  |  | **1** | **2** | **3** | **4** | **5** |
| 1 | #Accessions | 90 | 70 | 74 | 90 | 44 |
| 2 | Minimum | 202.8 | 201.9 | 224.4 | 191.1 | 91.9 |
| 3 | 25% Percentile | 229.9 | 240.9 | 249.6 | 230.1 | 187.2 |
| 4 | Median | 244.1 | 253.2 | 260.9 | 240.7 | 209.6 |
| 5 | 75% Percentile | 256.6 | 264.2 | 277.0 | 251.6 | 227.3 |
| 6 | Maximum | 287.9 | 292.9 | 337.8 | 291.9 | 245.1 |
| 7 | Mean | 243.2 | 253.4 | 265.8 | 241.0 | 203.3 |
| 8 | Std. Deviation | 20.4 | 19.9 | 22.8 | 18.2 | 33.2 |
| 9 | Std. Error of Mean | 2.2 | 2.4 | 2.6 | 1.9 | 5.0 |
| 10 | Lower 95% CI | 238.9 | 248.7 | 260.5 | 237.2 | 193.2 |
| 11 | Upper 95% CI | 247.4 | 258.1 | 271.0 | 244.8 | 213.4 |
| 12 | Range | 113.5 | 91 | 113.4 | 100.8 | 153.2 |
| 13 | CV | 8.856 | 7.839 | 8.569 | 7.532 | 16.33 |

Table S6 (Continued…)

| **No.** | **g. Panicle length (cm)** | **Cluster groups** | | | | |
| --- | --- | --- | --- | --- | --- | --- |
|  |  | **1** | **2** | **3** | **4** | **5** |
| 1 | #Accessions | 90 | 70 | 74 | 90 | 44 |
| 2 | Minimum | 37.3 | 34.1 | 43.8 | 42.3 | 26.2 |
| 3 | 25% Percentile | 50.4 | 51.4 | 59.0 | 54.6 | 34.0 |
| 4 | Median | 55.1 | 56.7 | 64.2 | 60.1 | 37.8 |
| 5 | 75% Percentile | 60.0 | 61.0 | 69.5 | 63.4 | 41.9 |
| 6 | Maximum | 67.5 | 72.4 | 88.1 | 80.9 | 47.8 |
| 7 | Mean | 54.6 | 56.1 | 64.6 | 59.3 | 37.8 |
| 8 | Std. Deviation | 7.2 | 8.1 | 8.7 | 7.1 | 5.1 |
| 9 | Std. Error of Mean | 0.8 | 1.0 | 1.0 | 0.7 | 0.8 |
| 10 | Lower 95% CI | 53.1 | 54.1 | 62.6 | 57.8 | 36.3 |
| 11 | Upper 95% CI | 56.1 | 58.0 | 66.6 | 60.8 | 39.4 |
| 12 | Range | 37.5 | 38.3 | 44.3 | 38.6 | 21.6 |
| 13 | CV | 17.97 | 14.39 | 13.54 | 11.9 | 13.46 |

| **No.** | **h. Tiller count**  **(plant^-1^)** | **Cluster groups** | | | | |
| --- | --- | --- | --- | --- | --- | --- |
|  |  | **1** | **2** | **3** | **4** | **5** |
| 1 | #Accessions | 90 | 70 | 74 | 90 | 44 |
| 2 | Minimum | 6.3 | 7.0 | 4.7 | 4.3 | 5.7 |
| 3 | 25% Percentile | 10.6 | 11.7 | 7.9 | 7.0 | 9.7 |
| 4 | Median | 12.3 | 14.0 | 9.7 | 9.2 | 11.7 |
| 5 | 75% Percentile | 14.7 | 15.7 | 10.4 | 10.8 | 13.9 |
| 6 | Maximum | 21.7 | 22.0 | 13.7 | 17.3 | 19.0 |
| 7 | Mean | 12.7 | 14.0 | 9.2 | 9.2 | 11.7 |
| 8 | Std. Deviation | 3.1 | 3.0 | 2.0 | 2.7 | 2.9 |
| 9 | Std. Error of Mean | 0.3 | 0.4 | 0.2 | 0.3 | 0.4 |
| 10 | Lower 95% CI | 12.1 | 13.3 | 8.8 | 8.7 | 10.8 |
| 11 | Upper 95% CI | 13.4 | 14.7 | 9.7 | 9.8 | 12.6 |
| 12 | Range | 13 | 15 | 9 | 13 | 13.3 |
| 13 | CV | 23.14 | 21.45 | 21.34 | 29.12 | 24.77 |

| **No.** | **i. Floret count**  **(spikelet^-1^)** | **Cluster groups** | | | | |
| --- | --- | --- | --- | --- | --- | --- |
|  |  | **1** | **2** | **3** | **4** | **5** |
| 1 | #Accessions | 90 | 70 | 74 | 90 | 44 |
| 2 | Minimum | 3.3 | 3.6 | 3.6 | 3.1 | 2.8 |
| 3 | 25% Percentile | 4.2 | 4.6 | 5.0 | 4.2 | 3.8 |
| 4 | Median | 4.6 | 4.9 | 5.6 | 4.7 | 4.4 |
| 5 | 75% Percentile | 4.9 | 5.2 | 6.4 | 5.3 | 5.1 |
| 6 | Maximum | 7.0 | 6.3 | 8.1 | 6.4 | 6.3 |
| 7 | Mean | 4.6 | 4.9 | 5.7 | 4.8 | 4.4 |
| 8 | Std. Deviation | 0.6 | 0.6 | 1.1 | 0.8 | 0.9 |
| 9 | Std. Error of Mean | 0.1 | 0.1 | 0.1 | 0.1 | 0.1 |
| 10 | Lower 95% CI | 4.4 | 4.8 | 5.4 | 4.6 | 4.1 |
| 11 | Upper 95% CI | 4.7 | 5.1 | 5.9 | 5.0 | 4.6 |
| 12 | Range | 4 | 2.7 | 4.5 | 3.3 | 3.5 |
| 13 | CV | 15.77 | 11.92 | 18.67 | 16.14 | 20.76 |

Table S6 (Continued…)

| **No.** | **j. HSW** | **Cluster groups** | | | | |
| --- | --- | --- | --- | --- | --- | --- |
|  |  | **1** | **2** | **3** | **4** | **5** |
| 1 | #Accessions | 90 | 70 | 74 | 90 | 44 |
| 2 | Minimum | 21.2 | 23.0 | 25.9 | 20.9 | 4.4 |
| 3 | 25% Percentile | 26.1 | 27.0 | 30.5 | 26.8 | 22.6 |
| 4 | Median | 28.3 | 30.6 | 32.4 | 28.7 | 24.1 |
| 5 | 75% Percentile | 30.5 | 34.7 | 34.3 | 31.4 | 25.2 |
| 6 | Maximum | 36.0 | 45.2 | 44.4 | 37.3 | 30.5 |
| 7 | Mean | 28.3 | 30.9 | 32.9 | 29.0 | 23.5 |
| 8 | Std. Deviation | 3.2 | 5.0 | 3.6 | 3.2 | 3.7 |
| 9 | Std. Error of Mean | 0.3 | 0.6 | 0.4 | 0.3 | 0.6 |
| 10 | Lower 95% CI | 27.7 | 29.7 | 32.1 | 28.3 | 22.4 |
| 11 | Upper 95% CI | 29.0 | 32.1 | 33.8 | 29.7 | 24.7 |
| 12 | Range | 14.8 | 22.2 | 18.5 | 16.4 | 26.1 |
| 13 | CV | 11.64 | 16.16 | 11.06 | 10.92 | 15.94 |

| **No.** | **k. Seed area (mm^2^)** | **Cluster groups** | | | | |
| --- | --- | --- | --- | --- | --- | --- |
|  |  | **1** | **2** | **3** | **4** | **5** |
| 1 | #Accessions | 90 | 70 | 74 | 90 | 44 |
| 2 | Minimum | 0.49 | 0.45 | 0.54 | 0.36 | 0.18 |
| 3 | 25% Percentile | 0.57 | 0.58 | 0.62 | 0.58 | 0.53 |
| 4 | Median | 0.61 | 0.65 | 0.65 | 0.62 | 0.57 |
| 5 | 75% Percentile | 0.65 | 0.69 | 0.68 | 0.65 | 0.60 |
| 6 | Maximum | 0.76 | 0.93 | 0.81 | 0.89 | 0.74 |
| 7 | Mean | 0.61 | 0.64 | 0.66 | 0.61 | 0.55 |
| 8 | Std. Deviation | 0.06 | 0.09 | 0.05 | 0.07 | 0.09 |
| 9 | Std. Error of Mean | 0.01 | 0.01 | 0.01 | 0.01 | 0.01 |
| 10 | Lower 95% CI | 0.60 | 0.62 | 0.65 | 0.60 | 0.53 |
| 11 | Upper 95% CI | 0.62 | 0.66 | 0.67 | 0.63 | 0.58 |
| 12 | Range | 0.24 | 0.48 | 0.27 | 0.53 | 0.56 |
| 13 | CV | 7.142 | 13.53 | 7.724 | 11.88 | 15.62 |

**Table S7. Pair-wise comparison of effects of cluster groups based upon 11 quantitative agronomic traits of *E. tef* to investigate the statistical significance of cluster groups.** The traits considered were a) fresh weight, b) dry weight, c) seed yield, d) straw yield, e) harvest index, f) plant height, g) panicle length, h) tiller count, i) floret count, j) hundred-seed weight, and k) seed area. The difference between any two pair of panicle means was declared at *P*<0.0001 (****); P<0.001 (***); P<0.01 (**); P<0.1 (*); ns, not significant. SE, standard error.

| **a. Fresh weight (g plant^-1^)** | | | | | | |
| --- | --- | --- | --- | --- | --- | --- |
| **No.** | **Comparisons** | **Mean1** | **Mean2** | **SE of diff.** | **Summary** | **Adj. P value** |
| 1 | Cluster 1 vs. cluster 2 | 133.4 | 175.3 | 3.729 | **** | <0.0001 |
| 2 | Cluster 1 vs. cluster 3 | 133.4 | 141.0 | 3.672 | ns | 0.2447 |
| 3 | Cluster 1 vs. cluster 4 | 133.4 | 95.8 | 3.488 | **** | <0.0001 |
| 4 | Cluster 1 vs. cluster 5 | 133.4 | 94.6 | 4.304 | **** | <0.0001 |
| 5 | Cluster 2 vs. cluster 3 | 175.3 | 141.0 | 3.901 | **** | <0.0001 |
| 6 | Cluster 2 vs. cluster 4 | 175.3 | 95.8 | 3.729 | **** | <0.0001 |
| 7 | Cluster 2 vs. cluster 5 | 175.3 | 94.6 | 4.501 | **** | <0.0001 |
| 8 | Cluster 3 vs. cluster 4 | 141.0 | 95.8 | 3.672 | **** | <0.0001 |
| 9 | Cluster 3 vs. cluster 5 | 141.0 | 94.6 | 4.454 | **** | <0.0001 |
| 10 | Cluster 4 vs. cluster 5 | 95.8 | 94.6 | 4.304 | ns | 0.9987 |

| **b. Dry weight (g plant^-1^)** | | | | | | |
| --- | --- | --- | --- | --- | --- | --- |
| **No.** | **Comparisons** | **Mean1** | **Mean2** | **SE of diff.** | **Summary** | **Adj. P value** |
| 1 | Cluster 1 vs. cluster 2 | 54.68 | 76.46 | 1.518 | **** | <0.0001 |
| 2 | Cluster 1 vs. cluster 3 | 54.68 | 56.59 | 1.495 | ns | 0.7027 |
| 3 | Cluster 1 vs. cluster 4 | 54.68 | 36.71 | 1.42 | **** | <0.0001 |
| 4 | Cluster 1 vs. cluster 5 | 54.68 | 35.46 | 1.752 | **** | <0.0001 |
| 5 | Cluster 2 vs. cluster 3 | 76.46 | 56.59 | 1.588 | **** | <0.0001 |
| 6 | Cluster 2 vs. cluster 4 | 76.46 | 36.71 | 1.518 | **** | <0.0001 |
| 7 | Cluster 2 vs. cluster 5 | 76.46 | 35.46 | 1.832 | **** | <0.0001 |
| 8 | Cluster 3 vs. cluster 4 | 56.59 | 36.71 | 1.495 | **** | <0.0001 |
| 9 | Cluster 3 vs. cluster 5 | 56.59 | 35.46 | 1.813 | **** | <0.0001 |
| 10 | Cluster 4 vs. cluster 5 | 36.71 | 35.46 | 1.752 | ns | 0.9531 |

| **c. Seed yield** **(g plant^-1^)** | | | | | | |
| --- | --- | --- | --- | --- | --- | --- |
| **No.** | **Comparisons** | **Mean1** | **Mean2** | **SE of diff.** | **Summary** | **Adj. P value** |
| 1 | Cluster 1 vs. cluster 2 | 10.7 | 15.0 | 0.4060 | **** | <0.0001 |
| 2 | Cluster 1 vs. cluster 3 | 10.7 | 8.5 | 0.3997 | **** | <0.0001 |
| 3 | Cluster 1 vs. cluster 4 | 10.7 | 7.3 | 0.3797 | **** | <0.0001 |
| 4 | Cluster 1 vs. cluster 5 | 10.7 | 5.5 | 0.4686 | **** | <0.0001 |
| 5 | Cluster 2 vs. cluster 3 | 15.0 | 8.5 | 0.4247 | **** | <0.0001 |
| 6 | Cluster 2 vs. cluster 4 | 15.0 | 7.3 | 0.4060 | **** | <0.0001 |
| 7 | Cluster 2 vs. cluster 5 | 15.0 | 5.5 | 0.4901 | **** | <0.0001 |
| 8 | Cluster 3 vs. cluster 4 | 8.5 | 7.3 | 0.3997 | * | 0.0259 |
| 9 | Cluster 3 vs. cluster 5 | 8.5 | 5.5 | 0.4849 | **** | <0.0001 |
| 10 | Cluster 4 vs. cluster 5 | 7.3 | 5.5 | 0.4686 | ** | 0.0014 |

Table S7 (Continued…)

| **d. Straw yield (g plant^-1^)** | | | | | | |
| --- | --- | --- | --- | --- | --- | --- |
| **No.** | **Comparisons** | **Mean1** | **Mean2** | **SE of diff.** | **Summary** | **Adj. P value** |
| 1 | Cluster 1 vs. cluster 2 | 44.0 | 61.5 | 1.384 | **** | <0.0001 |
| 2 | Cluster 1 vs. cluster 3 | 44.0 | 48.1 | 1.363 | * | 0.0219 |
| 3 | Cluster 1 vs. cluster 4 | 44.0 | 29.6 | 1.295 | **** | <0.0001 |
| 4 | Cluster 1 vs. cluster 5 | 44.0 | 29.7 | 1.598 | **** | <0.0001 |
| 5 | Cluster 2 vs. cluster 3 | 61.5 | 48.1 | 1.448 | **** | <0.0001 |
| 6 | Cluster 2 vs. cluster 4 | 61.5 | 29.6 | 1.384 | **** | <0.0001 |
| 7 | Cluster 2 vs. cluster 5 | 61.5 | 29.7 | 1.671 | **** | <0.0001 |
| 8 | Cluster 3 vs. cluster 4 | 48.1 | 29.6 | 1.363 | **** | <0.0001 |
| 9 | Cluster 3 vs. cluster 5 | 48.1 | 29.7 | 1.654 | **** | <0.0001 |
| 10 | Cluster 4 vs. cluster 5 | 29.6 | 29.7 | 1.598 | ns | >0.9999 |

| **e. Harvest index** | | | | | | |
| --- | --- | --- | --- | --- | --- | --- |
| **No.** | **Comparisons** | **Mean1** | **Mean2** | **SE of diff.** | **Summary** | **Adj. P value** |
| 1 | Cluster 1 vs. cluster 2 | 0.1947 | 0.1966 | 0.007051 | ns | 0.9988 |
| 2 | Cluster 1 vs. cluster 3 | 0.1947 | 0.1526 | 0.006943 | **** | <0.0001 |
| 3 | Cluster 1 vs. cluster 4 | 0.1947 | 0.1986 | 0.006595 | ns | 0.9766 |
| 4 | Cluster 1 vs. cluster 5 | 0.1947 | 0.1636 | 0.008139 | ** | 0.0015 |
| 5 | Cluster 2 vs. cluster 3 | 0.1966 | 0.1526 | 0.007377 | **** | <0.0001 |
| 6 | Cluster 2 vs. cluster 4 | 0.1966 | 0.1986 | 0.007051 | ns | 0.9986 |
| 7 | Cluster 2 vs. cluster 5 | 0.1966 | 0.1636 | 0.008512 | ** | 0.0012 |
| 8 | Cluster 3 vs. cluster 4 | 0.1526 | 0.1986 | 0.006943 | **** | <0.0001 |
| 9 | Cluster 3 vs. cluster 5 | 0.1526 | 0.1636 | 0.008423 | ns | 0.6826 |
| 10 | Cluster 4 vs. cluster 5 | 0.1986 | 0.1636 | 0.008139 | *** | 0.0002 |

| **f. Plant height (cm)** | | | | | | |
| --- | --- | --- | --- | --- | --- | --- |
| **No.** | **Comparisons** | **Mean1** | **Mean2** | **SE of diff.** | **Summary** | **Adj. P value** |
| 1 | Cluster 1 vs. cluster 2 | 243.2 | 253.4 | 3.538 | * | 0.0326 |
| 2 | Cluster 1 vs. cluster 3 | 243.2 | 265.8 | 3.484 | **** | <0.0001 |
| 3 | Cluster 1 vs. cluster 4 | 243.2 | 241.0 | 3.309 | ns | 0.9684 |
| 4 | Cluster 1 vs. cluster 5 | 243.2 | 203.3 | 4.084 | **** | <0.0001 |
| 5 | Cluster 2 vs. cluster 3 | 253.4 | 265.8 | 3.701 | ** | 0.0081 |
| 6 | Cluster 2 vs. cluster 4 | 253.4 | 241.0 | 3.538 | ** | 0.0048 |
| 7 | Cluster 2 vs. cluster 5 | 253.4 | 203.3 | 4.271 | **** | <0.0001 |
| 8 | Cluster 3 vs. cluster 4 | 265.8 | 241.0 | 3.484 | **** | <0.0001 |
| 9 | Cluster 3 vs. cluster 5 | 265.8 | 203.3 | 4.226 | **** | <0.0001 |
| 10 | Cluster 4 vs. cluster 5 | 241.0 | 203.3 | 4.084 | **** | <0.0001 |

| **g. Panicle length (cm)** | | | | | | |
| --- | --- | --- | --- | --- | --- | --- |
| **No.** | **Comparisons** | **Mean1** | **Mean2** | **SE of diff.** | **Summary** | **Adj. P value** |
| 1 | Cluster 1 vs. cluster 2 | 54.6 | 56.1 | 1.189 | ns | 0.7339 |
| 2 | Cluster 1 vs. cluster 3 | 54.6 | 64.6 | 1.171 | **** | <0.0001 |
| 3 | Cluster 1 vs. cluster 4 | 54.6 | 59.3 | 1.112 | *** | 0.0003 |
| 4 | Cluster 1 vs. cluster 5 | 54.6 | 37.8 | 1.373 | **** | <0.0001 |
| 5 | Cluster 2 vs. cluster 3 | 56.1 | 64.6 | 1.244 | **** | <0.0001 |
| 6 | Cluster 2 vs. cluster 4 | 56.1 | 59.3 | 1.189 | ns | 0.0528 |
| 7 | Cluster 2 vs. cluster 5 | 56.1 | 37.8 | 1.436 | **** | <0.0001 |
| 8 | Cluster 3 vs. cluster 4 | 64.6 | 59.3 | 1.171 | **** | <0.0001 |
| 9 | Cluster 3 vs. cluster 5 | 64.6 | 37.8 | 1.421 | **** | <0.0001 |
| 10 | Cluster 4 vs. cluster 5 | 59.3 | 37.8 | 1.373 | **** | <0.0001 |

Table S7 (Continued …)

| **h. Tiller count (plant^-1^)** | | | | | | |
| --- | --- | --- | --- | --- | --- | --- |
| **No.** | **Comparisons** | **Mean1** | **Mean2** | **SE of diff.** | **Summary** | **Adj. P value** |
| 1 | Cluster 1 vs. cluster 2 | 12.7 | 14.0 | 0.4386 | * | 0.045 |
| 2 | Cluster 1 vs. cluster 3 | 12.7 | 9.2 | 0.4319 | **** | <0.0001 |
| 3 | Cluster 1 vs. cluster 4 | 12.7 | 9.2 | 0.4103 | **** | <0.0001 |
| 4 | Cluster 1 vs. cluster 5 | 12.7 | 11.7 | 0.5063 | ns | 0.2625 |
| 5 | Cluster 2 vs. cluster 3 | 14.0 | 9.2 | 0.4589 | **** | <0.0001 |
| 6 | Cluster 2 vs. cluster 4 | 14.0 | 9.2 | 0.4386 | **** | <0.0001 |
| 7 | Cluster 2 vs. cluster 5 | 14.0 | 11.7 | 0.5295 | *** | 0.0003 |
| 8 | Cluster 3 vs. cluster 4 | 9.2 | 9.2 | 0.4319 | ns | >0.9999 |
| 9 | Cluster 3 vs. cluster 5 | 9.2 | 11.7 | 0.5239 | **** | <0.0001 |
| 10 | Cluster 4 vs. cluster 5 | 9.2 | 11.7 | 0.5063 | **** | <0.0001 |

| **i. Floret count (spikelet^-1^)** | | | | | | |
| --- | --- | --- | --- | --- | --- | --- |
| **No.** | **Comparisons** | **Mean1** | **Mean2** | **SE of diff.** | **Summary** | **Adj. P value** |
| 1 | Cluster 1 vs. cluster 2 | 4.6 | 4.9 | 0.1271 | * | 0.0456 |
| 2 | Cluster 1 vs. cluster 3 | 4.6 | 5.7 | 0.1252 | **** | <0.0001 |
| 3 | Cluster 1 vs. cluster 4 | 4.6 | 4.8 | 0.1189 | ns | 0.316 |
| 4 | Cluster 1 vs. cluster 5 | 4.6 | 4.4 | 0.1468 | ns | 0.644 |
| 5 | Cluster 2 vs. cluster 3 | 4.9 | 5.7 | 0.1330 | **** | <0.0001 |
| 6 | Cluster 2 vs. cluster 4 | 4.9 | 4.8 | 0.1271 | ns | 0.8586 |
| 7 | Cluster 2 vs. cluster 5 | 4.9 | 4.4 | 0.1535 | ** | 0.0032 |
| 8 | Cluster 3 vs. cluster 4 | 5.7 | 4.8 | 0.1252 | **** | <0.0001 |
| 9 | Cluster 3 vs. cluster 5 | 5.7 | 4.4 | 0.1519 | **** | <0.0001 |
| 10 | Cluster 4 vs. cluster 5 | 4.8 | 4.4 | 0.1468 | * | 0.0304 |

| **j. Hundred-seed weight** | | | | | | |
| --- | --- | --- | --- | --- | --- | --- |
| **No.** | **Comparisons** | **Mean1** | **Mean2** | **SE of diff.** | **Summary** | **Adj. P value** |
| 1 | Cluster 1 vs. cluster 2 | 28.3 | 30.9 | 0.5958 | *** | 0.0002 |
| 2 | Cluster 1 vs. cluster 3 | 28.3 | 32.9 | 0.5867 | **** | <0.0001 |
| 3 | Cluster 1 vs. cluster 4 | 28.3 | 29.0 | 0.5573 | ns | 0.7593 |
| 4 | Cluster 1 vs. cluster 5 | 28.3 | 23.5 | 0.6877 | **** | <0.0001 |
| 5 | Cluster 2 vs. cluster 3 | 30.9 | 32.9 | 0.6233 | ** | 0.0096 |
| 6 | Cluster 2 vs. cluster 4 | 30.9 | 29.0 | 0.5958 | * | 0.0131 |
| 7 | Cluster 2 vs. cluster 5 | 30.9 | 23.5 | 0.7193 | **** | <0.0001 |
| 8 | Cluster 3 vs. cluster 4 | 32.9 | 29.0 | 0.5867 | **** | <0.0001 |
| 9 | Cluster 3 vs. cluster 5 | 32.9 | 23.5 | 0.7117 | **** | <0.0001 |
| 10 | Cluster 4 vs. cluster 5 | 29.0 | 23.5 | 0.6877 | **** | <0.0001 |

| **k. Seed area (mm^2^)** | | | | | | |
| --- | --- | --- | --- | --- | --- | --- |
| **No.** | **Comparisons** | **Mean1** | **Mean2** | **SE of diff.** | **Summary** | **Adj. P value** |
| 1 | Cluster 1 vs. cluster 2 | 0.61 | 0.64 | 0.01128 | * | 0.0273 |
| 2 | Cluster 1 vs. cluster 3 | 0.61 | 0.66 | 0.01111 | *** | 0.0002 |
| 3 | Cluster 1 vs. cluster 4 | 0.61 | 0.61 | 0.01055 | ns | 0.9961 |
| 4 | Cluster 1 vs. cluster 5 | 0.61 | 0.55 | 0.01302 | *** | 0.0002 |
| 5 | Cluster 2 vs. cluster 3 | 0.64 | 0.66 | 0.01181 | ns | 0.6984 |
| 6 | Cluster 2 vs. cluster 4 | 0.64 | 0.61 | 0.01128 | ns | 0.0705 |
| 7 | Cluster 2 vs. cluster 5 | 0.64 | 0.55 | 0.01362 | **** | <0.0001 |
| 8 | Cluster 3 vs. cluster 4 | 0.66 | 0.61 | 0.01111 | *** | 0.0007 |
| 9 | Cluster 3 vs. cluster 5 | 0.66 | 0.55 | 0.01348 | **** | <0.0001 |
| 10 | Cluster 4 vs. cluster 5 | 0.61 | 0.55 | 0.01302 | **** | <0.0001 |

**Table S8. Original data sets used to evaluate 368 *E. tef* accessions.** See attached Excel spread sheet.
